# Evaluation of Intensive vs Standard Blood Pressure Reduction and Association With Cognitive Decline and Dementia: A Systematic Review and Meta-analysis

**DOI:** 10.1001/jamanetworkopen.2021.34553

**Published:** 2021-11-22

**Authors:** Caroline Dallaire-Théroux, Marie-Hélène Quesnel-Olivo, Karine Brochu, Frédéric Bergeron, Sarah O’Connor, Alexis F. Turgeon, Robert Jr Laforce, Steve Verreault, Marie-Christine Camden, Simon Duchesne

**Affiliations:** 1 Division of Neuroscience, Hôpital de l’Enfant-Jésus, Centre Hospitalier Universitaire (CHU) de Québec-Université Laval, Québec City, Québec, Canada; 2 CERVO Brain Research Center, Centre intégré universitaire de santé et services sociaux de la Capitale Nationale, Québec City, Québec, Canada; 3 Clinique Interdisciplinaire de Mémoire, CHU de Québec-Université Laval, Québec City, Québec, Canada; 4 Université Laval Library, Québec City, Québec, Canada; 5 Faculty of Pharmacy, Institut universitaire de cardiologie et pneumologie de Québec (IUCPQ), Université Laval, Québec City, Québec, Canada; 6 Division of Critical Care Medicine, Department of Anesthesiology and Critical Care Medicine, Université Laval, Québec City, Québec, Canada; 7 CHU de Québec–Université Laval Research Center, Population Health and Optimal Health Practices Research Unit, Trauma–Emergency–Critical Care Medicine, Québec City, Québec, Canada; 8 Department of Radiology and Nuclear Medicine, Faculty of Medicine, Université Laval, Québec City, Québec, Canada

## Abstract

**Importance:**

Optimal blood pressure (BP) targets for the prevention of cognitive impairment remain uncertain.

**Objective:**

To explore the association of intensive (ie, lower than usual) BP reduction vs standard BP management with the incidence of cognitive decline and dementia in adults with hypertension.

**Data Sources and Study Selection:**

A systematic review and meta-analysis of randomized clinical trials that evaluated the association of intensive systolic BP lowering on cognitive outcomes by searching MEDLINE, Embase, CENTRAL, Web of Science, CINAHL, PsycINFO, the International Clinical Trials Registry Platform, and ClinicalTrials.gov from database inception to October 27, 2020.

**Data Extraction and Synthesis:**

Data screening and extraction were performed independently by 2 reviewers based on Preferred Reporting Items for Systematic Reviews and Meta-analyses guidelines. The risk of bias was assessed using the Cochrane risk of bias 2 tool. Random-effects models with the inverse variance method were used for pooled analyses. The presence of potential heterogeneity was evaluated with the *I*^2^ index.

**Main Outcomes and Measures:**

The primary outcome was cognitive decline. Secondary outcomes included the incidence of dementia, mild cognitive impairment (MCI), cerebrovascular events, serious adverse events, and all-cause mortality.

**Results:**

From 7755 citations, we identified 16 publications from 5 trials with 17 396 participants (mean age, 65.7 years [range, 63.0-80.5 years]; 10 562 [60.5%] men) and 2 additional ongoing trials. All 5 concluded trials included in quantitative analyses were considered at unclear to high risk of bias. The mean follow-up duration was 3.3 years (range, 2.0 to 4.7 years). Intensive BP reduction was not significantly associated with global cognitive performance (standardized mean difference, 0.01; 95% CI, −0.04 to 0.06; *I*^2^ = 0%; 4 trials; 5246 patients), incidence of dementia (risk ratio [RR], 1.09; 95% CI, 0.32 to 3.67; *I*^2^ = 27%; 2 trials; 9444 patients) or incidence of MCI (RR, 0.91; 95% CI, 0.73 to 1.14; *I*^2^ = 74%; 2 trials; 10 774 patients) when compared with standard treatment. However, a reduction of cerebrovascular events in the intensive group was found (RR, 0.79; 95% CI, 0.67 to 0.93; *I*^2^ = 0%; 5 trials; 17 396 patients) without an increased risk of serious adverse events or mortality.

**Conclusions and Relevance:**

In this study, there was no significant association between BP reduction and lower risk of cognitive decline, dementia, or MCI. The certainty of this evidence was rated low because of the limited sample size, the risk of bias of included trials, and the observed statistical heterogeneity. Therefore, current available evidence does not justify the use of lower BP targets for the prevention of cognitive decline and dementia.

## Key Points

**Question:**

Is intensive blood pressure reduction associated with lower rates of cognitive decline and dementia?

**Findings:**

In this systematic review and meta-analysis of 5 randomized clinical trials with 17 396 participants, there was no significant association of lower blood pressure targets vs standard blood pressure management with the incidence of cognitive decline, dementia, and mild cognitive impairment in middle-aged and older adults with hypertension.

**Meaning:**

These findings suggest that current evidence does not support intensive blood pressure reduction as a preventive strategy for cognitive decline and dementia.

## Teaser

This systematic review and meta-analysis explores the association of intensive blood pressure reduction vs standard blood pressure management with the incidence of cognitive decline and dementia in adults with hypertension.

## Introduction

Dementia was declared a world health priority by the World Health Organization (WHO),^1^ with intense global research efforts dedicated toward the design of interventions to prevent, delay, or treat etiologies leading to cognitive impairment and dementia. Among those, cerebrovascular disease (CVD) is a major contributor.^2^ Indeed, an important overlap exists between CVD and neurodegenerative conditions, especially Alzheimer disease (AD), with more than half of autopsied cases being of mixed etiologies.^3^ CVD, AD, and mixed CVD/AD are associated with as many as 80% of all dementia cases in community-dwelling older persons.^4,5^

High blood pressure (BP) is an important risk factor shared by both CVD and AD.^6,7^ Considering that antihypertensive drugs are associated with a reduced risk of stroke,^8,9^ BP control can be viewed as a potential way to optimize brain health and reduce the global risk of dementia. Accordingly, a recent systematic review of randomized clinical trials^10^ found an association between BP reduction and reduced risk of cognitive decline. The WHO 2019 guidelines^11^ recommend that standard hypertension management be offered to adults with hypertension to reduce the risk of cognitive decline and/or dementia (very low quality of evidence, conditional strength of the recommendation).

Recently, lower BP targets were advocated for the prevention of mortality and vascular events in guidelines for high-risk populations with comorbid conditions, including coronary artery disease, previous stroke, heart failure, chronic kidney disease, chronic obstructive pulmonary disease, and diabetes.^12,13^ Recent guidelines from dementia experts^14^ also support that a systolic BP target of less than 120 mm Hg should be considered when deciding on the intensity of antihypertensive therapy in middle-aged and older persons with hypertension. In a recent trial, it was suggested that such an approach could have an effect on the incidence of mild cognitive impairment (MCI).^15^ However, the optimal BP target for the prevention of cognitive decline remains controversial,^16,17^ and the question of whether more aggressive BP control with lower targets is associated with better cognitive outcomes compared with standard BP control is still unresolved.

We hypothesized that lower BP targets could provide additional benefits to cognitive health. To support this hypothesis, we conducted a systematic review with meta-analyses to evaluate the association of intensive vs standard BP reduction in adults with hypertension for the prevention of cognitive decline and dementia.

## Methods

### Study Design

Our systematic review and meta-analysis was conducted following the recommendations of the *Cochrane Handbook for Systematic Reviews of Interventions*.^18^ We reported our results following the Preferred Reporting Items for Systematic Reviews and Meta-Analyses (PRISMA) guidelines.^19^ The final protocol was registered on PROSPERO on November 30, 2020, prior to the beginning of the study (CRD42020218390).

### Eligibility Criteria

Randomized clinical trials comparing intensive BP control (ie, lower than usual systolic BP targets or ≤135 mm Hg) with standard of care for hypertension (ie, systolic BP targets of ≤140 mm Hg for most populations^20,21,22,23^) were included, regardless of the class, number, and dose of antihypertensive agents used to achieve this goal. Trials performed in human adults of middle and older ages (defined as individuals aged 40 years and older for at least 80% of the study population) with high BP and with or without history of cardiovascular or cerebrovascular events were considered for inclusion. All community-dwelling participants without dementia were considered, identified either as cognitively healthy or with MCI. Participants with MCI should have objective evidence of cognitive decline without significant impairment in activities of daily living. At least 1 year of follow-up and 1 prespecified outcome measure (as described later) had to be assessed for the study to meet inclusion criteria. No restriction was applied to language, years, or type of publication.

### Search Strategy

The search strategy (developed by C.D.T. and F.B.) included free and controlled vocabulary for the population, the intervention, and the cognitive outcomes. We used the validated Cochrane highly sensitive filter for Medline (Ovid) to identify randomized clinical trials and adapted it for other databases.^24^ An extensive and systematic literature search was performed through MEDLINE (Ovid), Embase (Embase.com), Cochrane Central Register of Controlled Trials (CENTRAL), Web of Science, CINAHL, and PsycINFO (Ovid) databases for articles published from database inception to October 27, 2020. International Clinical Trials Registry Platform (ICTRP) and ClinicalTrials.gov were also searched for unpublished trials. Additional relevant citations were manually retrieved from reference lists of included trials and other published meta-analyses. The full search strategy is presented in eTable 1 in the Supplement.

### Study Selection and Data Extraction

Citations were downloaded to a reference manager software (EndNote version X9) and then uploaded to an online screening and extraction tool (Covidence). Two of 3 reviewers (C.D.T., M.H.Q.O., and K.B.) independently screened all identified titles and abstracts after duplicates were removed to select studies that potentially met the inclusion criteria. Full-text versions were then assessed to confirm eligibility. Any selection conflict was resolved by a fourth reviewer (M.C.C.). For each included trial, 2 of 3 reviewers (C.D.T., M.H.Q.O., and K.B.) independently extracted data using a standardized form that was previously piloted. Extracted data included study characteristics, baseline demographic characteristics (including self-reported sex at birth and ethnicity), and cognitive status of participants; description of the intervention and control groups; mean change in BP; duration of follow-up; and summary of reported outcome measures. Discrepancies were resolved through discussion, or when necessary, a fourth reviewer was consulted (M.C.C.).

### Outcome Measures

Our primary outcome was the incidence of cognitive decline (mean change in global cognitive function test scores within the study period). Secondary outcomes included incidence of probable dementia (any diagnostic criteria), incidence of MCI, incidence of cerebrovascular events (including ischemic and hemorrhagic strokes), serious adverse effects potentially attributable to antihypertensive therapy (such as falls, orthostatic hypotension, severe hypotension, and kidney failure), and all-cause mortality.

### Risk-of-Bias Assessment

The risk of bias of included trials was evaluated independently by 2 of 3 reviewers (C.D.T., M.H.Q.O., and K.B.) using the second version of the Cochrane risk-of-bias tool.^18^ Trials were assessed for each outcome on the following domains: bias arising from the randomization process, bias due to deviations from intended interventions, bias due to missing outcome data, bias in outcome measurement, and bias in selection of the reported result. An overall risk-of-bias judgement was reached for individual trials regarding each specific outcome. Disagreements were resolved by discussion or by a fourth reviewer (M.C.C.) in unsolved cases.

### Quality of Evidence

The quality of the evidence was evaluated for each outcome according to the Grades of Recommendation, Assessment, Development and Evaluation (GRADE) system (McMaster University).^25^ We graded the evidence on a scale ranging from very low (very uncertain about the estimate of clinical effect) to high (further research is unlikely to change the confidence in the estimated clinical effect).

### Statistical Analysis

Quantitative data were entered into RevMan version 5 (The Nordic Cochrane Center) for conducting our pooled analyses using random-effect models with the inverse variance method. Pooled estimates were presented as risk ratios (RRs) with 95% CIs for dichotomous data and as mean differences (or standardized mean differences [SMDs] if the same outcome was measured with different scales) with 95% CIs for continuous data. We assessed the presence of potential statistical heterogeneity with *I*^2^ statistical tests (0%-40% indicating that heterogeneity might not be important; 30%-60%, may represent moderate heterogeneity; 50%-90%, may represent substantial heterogeneity; and 75%-100%, considerable heterogeneity).^18^ We planned subgroup analyses based on the duration of follow-up (≤3 vs >3 years), age (<65 years vs >65 years), diabetic status, primary vs secondary prevention of cognitive decline, primary vs secondary prevention of stroke, and the risk of bias. We planned exploration of potential publication bias using funnel plots when 10 or more trials were available for a given outcome. Considering that only 2 studies were included in the analysis for incidence of dementia and that sample sizes were unbalanced,^26^ we performed a sensitivity analysis a posteriori using a fixed-effect model. A 95% CI excluding the value 1 for risk ratios and the value 0 for standardized mean differences was defined to determine statistical significance.

## Results

### Study Identification and Selection

**Figure 1.**
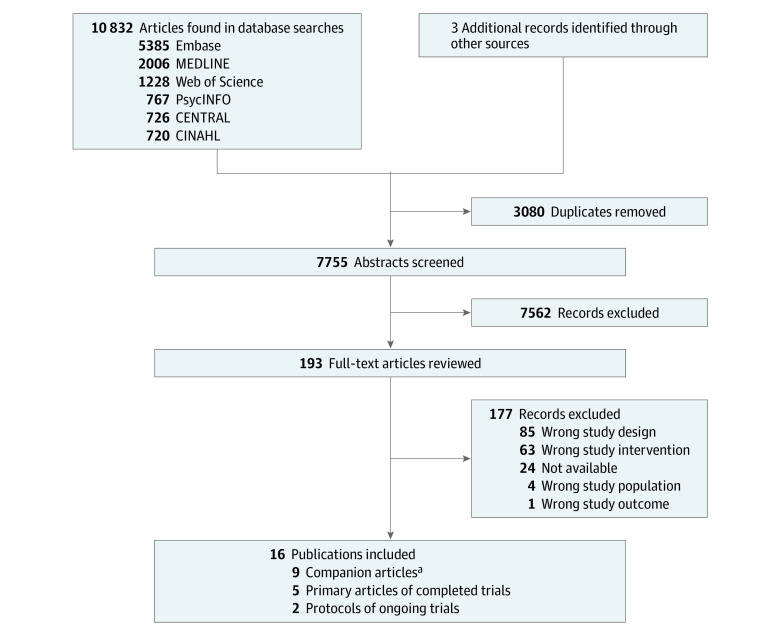
Study Flow Diagram ^a^Companion articles represent additional reports of published analyses involving the same study population.

Overall, our search yielded 10 835 citations, of which 7755 were screened after duplicate removal (Figure 1). Five randomized clinical trials (ACCORD BP,^27,28^ SPS3,^29,30^ SPRINT,^15,31,32,33,34^ PODCAST,^35,36,37^ and INFINITY^38,39^) from 14 publications and 2 protocols from ongoing and upcoming trials (ESH-CHL-SHOT^40^ and IBIS^41^) met eligibility criteria for inclusion.

### Characteristics of Included Studies

The details of the 7 selected trials are presented in Table 1, and baseline characteristics of participants from the 5 trials included in our quantitative analyses are found in eTable 2 in the Supplement. The total number of participants was 17 396 (intensive BP reduction, 8681; standard BP reduction, 8715). The mean follow-up was 3.3 years (range, 2.0 to 4.7 years). Combined studies included more men (10 562 [60.9%]) than women, with mostly White participants (10 060 [57.8%]) with a mean age of 65.7 years (range, 63.0 to 80.5 years). All included studies were prospective randomized open blinded end point (PROBE) trials comparing 2 different (ie, lower vs standard) systolic BP targets, with data analyzed on an intention-to-treat basis. Two trials met our eligibility criteria but could not be included in our pooled analyses. One trial was completed but still unpublished,^40^ while the other is ongoing.^41^ Of the 5 trials included in our pooled analyses, 4 were multicentric.^27,29,32,37^ Most studies were conducted in North America, but 1 study also included participants from Latin America and Spain,^29^ and 1 was exclusively conducted in the United Kingdom.^37^ Four studies were funded by the US National Institutes of Health^27,29,32,39^ and 1 by the UK Alzheimer Society and Stroke Association.^37^

**Table 1.** Characteristics of Included Studies

Trial	Design and country	Population	Participants (No.)		Intensive BP target , mm Hg	Standard BP target, mm Hg	BP achieved in intensive group, SBP/DBP, mm Hg	BP achieved in standard group, SBP/DBP, mm Hg	Follow-up, y	Measured outcomes
			Intensive	Standard						
ACCORD BP,^27,28^ 2010	RCT; US and Canada	Middle-aged and older participants with type 2 diabetes at high risk of cardiovascular events	2362	2371	SBP <120	SBP <140	119.3/64.4	133.5/70.5	4.7	ACCORD BP, primary outcome First occurrence of major cardiovascular event Secondary outcomes Primary outcome with revascularization or hospitalization Major coronary disease events Nonfatal myocardial infarction Fatal or nonfatal stroke Death from any cause Death from cardiovascular causes Hospitalization or death due to heart failure ACCORD-MIND, primary outcome Digit Symbol Substitution Test (processing speed) Secondary outcomes Rey Auditory Verbal Learning Test (verbal memory) Modified Stroop Color-Word Test (executive function) Mini-Mental State Examination (global cognition) Physician’s Health Questionnaire (depression)
SPS3,^29,30^ 2013	RCT; North America, Latin America, and Spain	Patients aged ≥30 y with cerebral small vessel disease and lacunar stroke within 6 mo	1501	1519	SBP <130	SBP 130-149	126.7/69.1	137.4/74.8	3.7 (3.0 for cognitive outcomes)	Original study, primary outcomes Ischemic stroke Intracranial hemorrhage Disabling stroke with mRS ≥3 Fatal stroke Secondary outcomes Acute myocardial infarct Major vascular events Death Serious complications of hypotension Serious complications related to antihypertensive medications Secondary analysis of cognitive function, primary outcome Cognitive Abilities Screening Instrument (global cognition) Secondary outcomes California Verbal Learning Test short and long delay cued tests, free recall test, and discriminability (memory) WAIS-III block design, symbol search, and digit span tests Controlled Oral Word Association test Grooved pegboard test Clox test
SPRINT,^15,31,32,33,34^ 2015	RCT; US and Puerto Rico	Adults ≥50 y with hypertension but without diabetes or history of stroke	4678	4683	SBP <120	SBP <140	121.4/68.7	136.2/76.3	3.3	Original study, primary outcome Composite outcome of myocardial infarction, acute coronary syndrome not resulting in myocardial infarction, stroke, acute decompensated heart failure, or death from cardiovascular causes Secondary outcomes Individual components of the primary composite outcome Death from any cause Composite primary outcome or death from any cause Serious adverse events SPRINT MIND, primary outcome Occurrence of adjudicated probable dementia Secondary outcomes Adjudicated mild cognitive impairment Composite outcome of MCI or probable dementia
PODCAST,^35,36,37^ 2017	RCT; UK	Patients 3-7 mo post ischemic stroke or intracerebral hemorrhage	41	42	SBP <125	SBP <140	130.0/72.9	140.5/77.4	2.0	Primary outcome Addenbrooke Cognitive Examination–Revised (global cognition) Secondary outcomes MoCA (global cognition) Mini-Mental State Examination (global cognition) Stroop test Trail-Making Tests A and B Category fluency Telephone Interview for Cognition–Modified IQCODE Cognitive impairment and dementia Others: quality of life, mood, function, health resource utilization, vascular events, serious adverse events
INFINITY,^38,39^ 2019	RCT; US	Patients ≥75 y with hypertension and normal or mildly impaired mobility and cognition who have detectable cerebrovascular disease	99	100	SBP ≤130	SBP ≤145	127.7/64.6	144.0/72.3	3.0	Primary outcomes Change from baseline in mobility parameters (gait times) Damage to brain white matter as demonstrated by accrual WMH volume and changes in diffusion tensor imaging Secondary outcomes Change from baseline in cognitive function (executive function, processing speed and memory) Safety end points (mortality, major nonfatal cardiovascular events, events of special interest potentially related to hypotension, including syncope and falls)
ESH-CHL-SHOT,^40^ 2020^a^	RCT; Europe and China	Patients ≥65 y with hypertension and stroke or transient ischemic attack 1 to 6 months prior to randomization	NA	NA	SBP <135-125, average 130; SBP <125, average 120	SBP <145-135, average 140	NA	NA	4.0	Primary outcome Time to occurrence of (recurrent) stroke (fatal and nonfatal) Secondary cardiovascular outcomes Time to first major cardiovascular event (composite outcome of cardiovascular death, nonfatal stroke, nonfatal myocardial infarction, vascular interventions, hospitalized heart failure) Coronary heart disease events (composite outcome of sudden death, fatal and nonfatal myocardial infarction, unstable angina, coronary interventions) All-cause death Cardiovascular death Hospitalized heart failure New-onset atrial fibrillation Ischemic stroke Hemorrhagic stroke Composite of stroke and transient ischemic attack Secondary neurological outcomes Cognitive impairment (MoCA) Dementia Tertiary outcomes Disability (mRS) Level of daily living activities (Barthel index) Depression (15-item Geriatric Depression scale) Changes in organ damage (microalbuminuria, proteinuria, stage 3B chronic kidney disease, ECG left-ventricular hypertrophy, other ancillary measurements)
IBIS,^41^ July 2021^b^	RCT; US and China	Patients ≥40 y with a history of symptomatic MRI/CT–confirmed ischemic stroke (3-12 months since last acute onset) and hypertension	NA	NA	SBP <120	SBP <140	NA	NA	4.0	Primary outcome Total recurrent stroke Secondary outcomes Major cardiovascular disease events (composite outcome of myocardial infarction, non–myocardial infarction acute coronary syndrome, stroke, hospitalized or treated heart failure, and cardiovascular disease deaths) Individual cardiovascular disease events All-cause mortality Cognitive decline and all-cause dementia Health-related quality of life Adverse events

Abbreviations: BP, blood pressure; CT, computed tomography; ECG, electrocardiogram; DBP, diastolic blood pressure; IQCODE, Informant Questionnaire on Cognitive Decline in the Elderly; MoCA, Montreal Cognitive Assessment; MRI, magnetic resonance imaging; mRS, modified Rankin Scale; NA, not applicable; RCT, randomized controlled trial; SBP, systolic blood pressure; WAIS, Wechsler Adult Intelligence Scale; WMH, white matter hyperintensities.

^a^ Results for this trial not yet available. Trial was terminated early due to insufficient patient recruitment and funding limitation.^42^

^b^ July 2021 estimated start date; results not yet available.

### Risk-of-Bias Assessment

**Figure 2.**
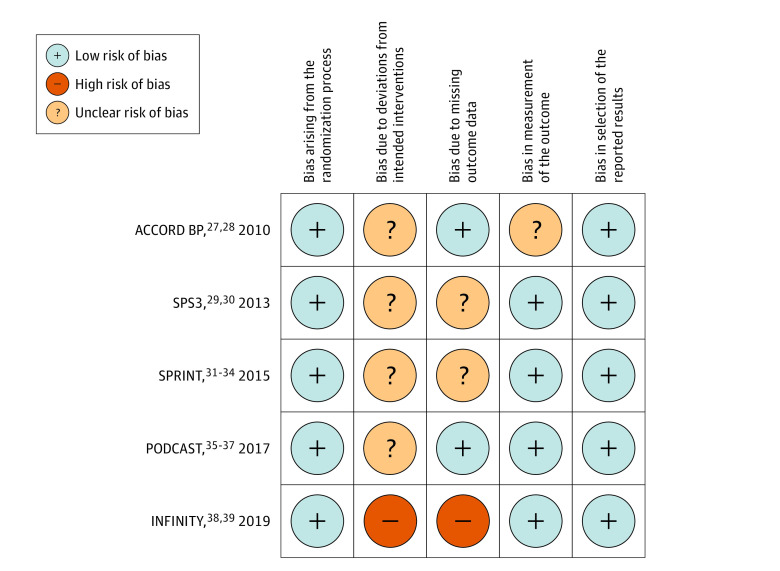
Risk of Bias Summary

The summary of the risk-of-bias assessment for each study is presented in Figure 2. Judgement was based on both published and unpublished data. The overall risk of bias was unclear for 4 studies^27,29,32,37^ and high for 1 study^39^ included in our meta-analysis. Because participants and clinicians of all included trials were unblinded to BP targets, we considered that there was unclear risk of bias due to deviations from intended interventions. The main concern regarding the missing outcome data was premature discontinuation from the study that could be potentially related to both the intervention group (adverse effects of intensive BP reduction) and the cognitive status (more cognitively impaired individuals).

### Primary Outcome: Cognitive Decline

**Figure 3.**
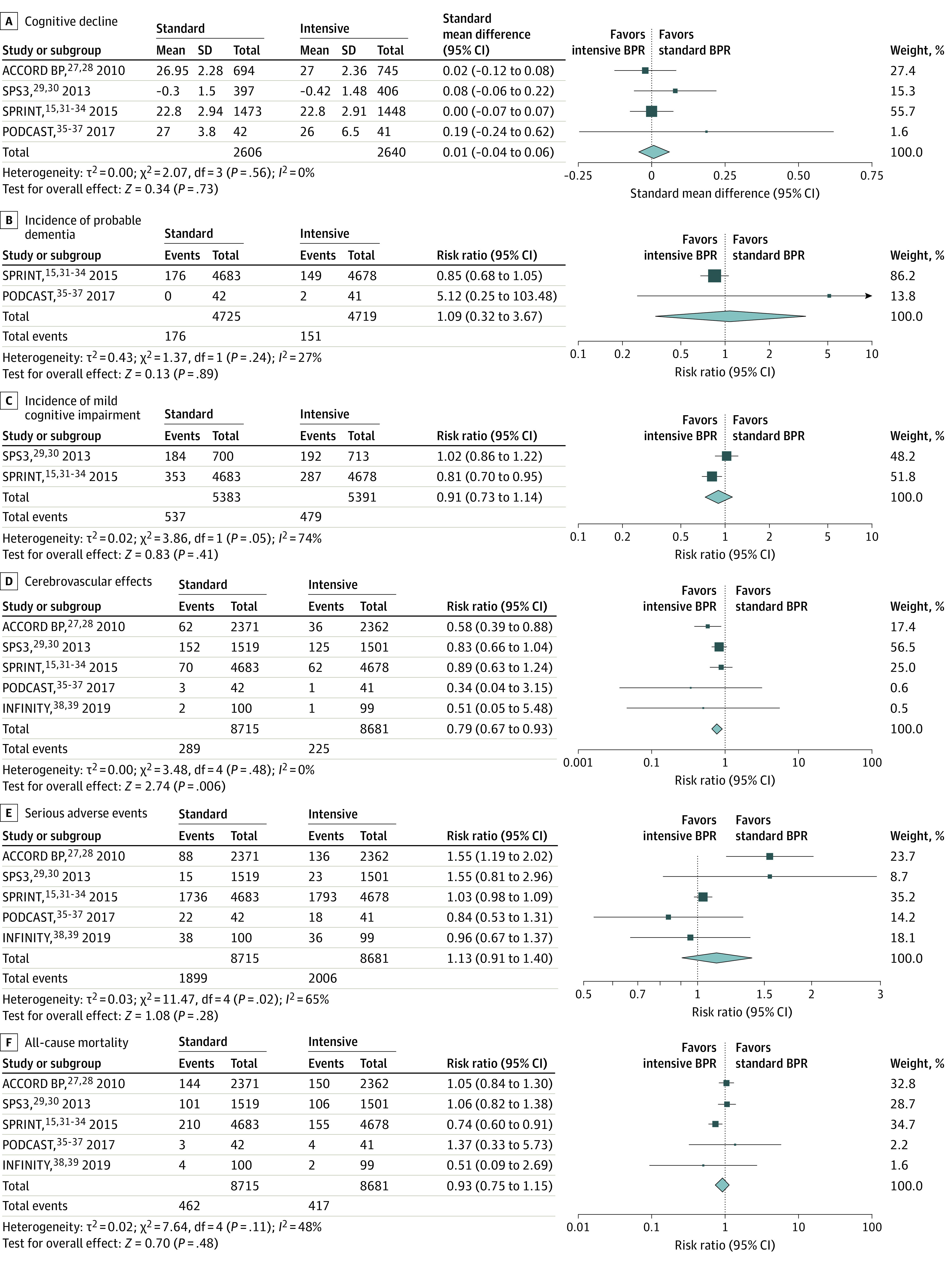
Association of Intensive vs Standard Blood Pressure Reduction (BPR) on Primary and Secondary Outcomes

Four studies^28,30,33,37^ provided data on cognitive decline, including a total of 5246 participants and a mean follow-up of 3.4 years (range, 2.0-4.7 years). Measurement of global cognitive function change from baseline was reported on the Mini-Mental State Examination in ACCORD BP^28^ and PODCAST,^37^ on the Cognitive Abilities Screening Instrument in SPS3,^30^ and on the Montreal Cognitive Assessment in SPRINT.^15^ Available data did not allow the direct transformation of scores on a same validated scale. Therefore, effect size estimates are reported as SMDs. Intensive compared with standard BP reduction was not associated with differential rates of cognitive decline (SMD, 0.01; 95% CI, −0.04 to 0.06; *I*^2^ = 0%) (Figure 3A), and this finding was consistent for all subgroup analyses, including stratification by study overall risk of bias (eTable 3 in the Supplement). Because of the insufficient number of trials (ie, <10) reporting on cognitive decline, we could not conclude on the presence of publication bias. Given that most trials were considered to be of unclear risk of bias and that results relied on surrogate outcomes of patient cognitive and functional status, we downgraded the quality of evidence by 2 levels. Thus, we graded the overall strength of evidence for an association with cognitive decline as low (Table 2).

**Table 2.** Summary of Findings: Intensive vs Standard Blood Pressure Reduction for Primary and Secondary Outcomes

Outcomes	Trials, No.	No. of events/total No. of participants		Effect size estimate (95% CI)	*I*^2^, %	Quality of the evidence (GRADE)
		Intensive	Standard			
Cognitive decline^a^	4	NA/2640	NA/2606	SMD, 0.01 (–0.04 to 0.06)	0	Low^b^
Incidence of probable dementia	2	151/4719	176/4725	RR, 1.09 (0.32 to 3.67)	27	Low^b^
Incidence of MCI	2	479/5391	537/5383	RR, 0.91 (0.73 to 1.14)	74	Low^c^
Cerebrovascular events	5	225/8681	289/8715	RR, 0.79 (0.67 to 0.93)	0	Moderate^d^
Serious adverse events	5	2006/8681	1899/8715	RR, 1.13 (0.91 to 1.40)	65	Low^c^
All-cause mortality	5	417/8681	462/8715	RR, 0.93 (0.75 to 1.15)	48	Low^c^

Abbreviations: MCI, mild cognitive impairment; NA, not applicable; RR, risk ratio; SMD, standardized mean difference.

^a^ There are no events for this category because it was measured on a quantitative scale.

^b^ Downgraded by 2 levels owing to unclear to high risk of bias of included studies and indirectness of evidence.

^c^ Downgraded by 2 levels owing to unclear to high risk of bias of included studies and significant heterogeneity.

^d^ Downgraded by 1 level owing to unclear to high risk of bias of included studies.

### Secondary Outcomes

#### Incidence of Probable Dementia

Two trials^15,37^ provided data on incident dementia, which included a total of 327 among 9444 participants (3.5%) diagnosed with probable dementia during a mean follow-up period of 2.7 years (range, 2.0-3.3 years). Because the 95% CI included the value 1, the risk of probable dementia did not significantly differ with intensive compared with standard BP reduction (RR, 1.09; 95% CI, 0.32-3.67; *I*^2^ = 27%) (Figure 3B and eTable 4 in the Supplement). Similarly, results from a sensitivity analysis using fixed-effect model showed no significant benefit with intensive interventions (RR, 0.86; 95% CI, 0.69-1.06) (eFigure in the Supplement). We graded the quality of the evidence for incidence of probable dementia as low owing to the risk of bias of included studies and indirectness of evidence related to their small number (Table 2).

#### Incidence of MCI

The incidence of MCI was reported in 2 trials^15,30^ of unclear risk of bias that included a total of 10 774 participants. By the end of the trials, 1016 participants (9.4%) were diagnosed with MCI during a mean follow-up period of 3.5 years (range, 3.3-3.7 years). The risk of MCI did not significantly differ between intensive and standard BP reduction strategies (RR, 0.91; 95% CI, 0.73-1.14; *I*^2^ = 74%) (Figure 3C and eTable 5 in the Supplement). Potential sources of statistical heterogeneity could not be explored because of the limited number of trials. We assessed the incidence of MCI as providing low-quality evidence (Table 2).

#### Cerebrovascular Events

The association of intensive BP lowering treatment with all types of strokes were available from all 5 trials,^27,29,32,37,39^ which included a total of 17 396 participants and 514 cerebrovascular events. Intensive BP control was associated with a 21% reduction in the risk of cerebrovascular events compared with usual treatment (RR, 0.79; 95% CI, 0.67-0.93; *I*^2^ = 0%) (Figure 3D). Subgroup analyses suggested that stroke risk reduction might be more important in patients with diabetes (eTable 6 in the Supplement). Given that all studies represented an unclear to high risk of bias, we downgraded the quality of evidence for an association with cerebrovascular events as moderate (Table 2).

#### Serious Adverse Events

A total of 3905 serious adverse events, including angioedema, hypotension, bradycardia, syncope, fall, and kidney failure, occurred among the 17 396 participants recruited in the 5 trials.^27,29,32,37,39^ Because of the large 95% CI including the value 1, it is uncertain whether there was a difference in the risk of SAE between participants allocated intensive treatment of hypertension and those allocated standard treatment (RR, 1.13; 95% CI, 0.91-1.40; *I*^2^ = 65%) (Figure 3E). While it does not meet the threshold for statistical significance, an RR potentially as large as 1.40 for the incidence of SAE would be quite concerning. Subgroup analyses revealed that statistical heterogeneity was mainly explained by age group and diabetes status (eTable 7 in the Supplement). We considered this pooled estimate of low quality of evidence (Table 2).

#### All-Cause Mortality

All 17 396 participants from the 5 trials^27,29,32,37,39^ contributed to analyses of all-cause mortality. A total of 879 participants (5.5%) died of cardiovascular and noncardiovascular causes across all BP targets. We found no evidence of a difference in the risk of mortality between intensive and standard BP control strategies (RR, 0.93; 95% CI, 0.75-1.15; *I*^2^ = 48%) (Figure 3F). The quality of evidence was considered low (Table 2). The association of intensive BP control with all-cause mortality varied with age group, diabetes status, and previous history of stroke, which could possibly explain the observed statistical heterogeneity (eTable 8 in the Supplement).

## Discussion

### Summary of Results

In our systematic review, we observed no significant association of lower BP targets compared with standard BP management with reduced incidence of cognitive decline in middle-aged and older adults with hypertension. Similarly, we also observed no association with the risk of developing dementia or MCI. Our findings were consistent based on the duration of follow-up, age, diabetes status, previous cognitive impairment or stroke, and the risk of bias. However, fewer cerebrovascular events were observed with lower BP targets with no significant difference in the rate of severe adverse events or mortality.

### Evidence in Context

Several reviews focusing on standard BP control interventions were previously published.^10,43,44,45,46^ Despite conflicting results, the 2 most recent meta-analyses^10,45^ found consistent associations of BP reduction with reduced risk of dementia and cognitive decline. Negative findings from prior studies may be explained by older age of participants^43^ and inclusion of nonpharmacologic interventions.^44^ Unlike previous publications, however, our systematic review aimed to examine the effectiveness of lower than usual BP targets, with standard, or guideline-based, BP targets as comparator. Contrary to our hypothesis, antihypertensive treatment with both targets was associated with comparable rates of cognitive decline and incidence of MCI and dementia. In other words, our results suggest that aiming at lower BP targets is not associated with additional benefit beyond the recognized protective effect of standard antihypertensive therapy on cognitive health. Of note, the mean duration of follow-up of included studies was limited to 3.3 years, and thus, this period might be too short to accurately detect cognitive impairment associated with chronic subclinical CVD. We would venture that, if present, it is unlikely that an effect would be detectable a window shorter than 5 to 10 years. Other factors that could have limited our capacity to detect an association include the variability in BP targets in the intervention and the inclusion of heterogenous populations with comorbid conditions.

Also, similar to what has been observed in other neurodegenerative conditions such as AD,^47^ it is possible that if intensive BP interventions are to have a protective effect on cognitive function, such interventions would need to be implemented earlier in the disease course. Indeed, as stated in the 2020 report of the Lancet Commission on dementia,^48^ persistent midlife hypertension, defined as starting at age 40 years, is associated with increased risk of late-life dementia. However, trials included in our meta-analysis were mostly performed outside the therapeutic window of intervention, with mean ages older than 60 years. Thus, later life BP control, coupled with a short period of follow-up, could be associated with smaller observable association of the intervention with outcomes.

Our results are consistent with those of 2 recent meta-analyses^12,49^ that found intensive BP control was associated with a reduced incidence of stroke, without significant increased risk of total severe adverse events and mortality. Only a small absolute excess of severe hypotension was detected with intensive interventions (0.3% vs 0.1% per person-year).^12^ A network meta-analysis also found lower rates of strokes with lower BP targets.^50^ Previous results from a meta-analysis of prospective cohort studies found that both prevalent and incident strokes are strong risk factors for all-cause dementia and that an history of stroke was associated with the incidence of dementia in older individuals.^51^ Hence, by reducing the number of cerebrovascular events, we can hypothesize that the incidence of cognitive decline and dementia would also be reduced. The relatively short duration of follow-up of published trials may explain why we did not observe such results in our review.

With the exception of stroke risk reduction,^52^ other reviews did not report an association of more aggressive BP lowering strategies with a lower number of total cardiovascular events in adults with hypertension and overt cardiovascular disease^53^ and diabetes.^52^ Yet, these 2 high-risk groups are often targeted for more strict BP control for the prevention of global mortality and cardiovascular events according to current international hypertension guidelines.^13^ Most recommendations were based on evidence from either observational studies, post hoc analyses of trials designed for various purposes, or results from a single clinical trial. Differences in the inclusion criteria between reviews may also explain the observed inconsistencies in the literature.

Finally, it is important to note that while previous studies^12,49^ and ours have not observed an increased risk of serious adverse events, it cannot also be excluded. These findings should raise caution on potential type II error for the risk of serious adverse events.

### Limitations

This study has limitations. First, we used controlled and free vocabulary related to cognitive outcomes in the search strategy. Hence, there is a risk that we missed important studies looking at secondary outcomes, such as cerebrovascular events, serious adverse events, and mortality. Second, we observed considerable variations among trials on the assessment of cognitive function; the use of different scales and follow-up intervals may have limited our ability to optimally evaluate a potential effect. Third, moderate to substantial residual statistical heterogeneity was observed in most analyses of secondary outcomes, limiting the interpretation of pooled estimates. Moreover, only 2 trials with unbalanced sample sizes were included for the analysis on incident dementia. Despite conducting a sensitivity analysis using a fixed-effect model, our analysis was not sufficiently robust to make a firm conclusion. Additionally, our results are possibly limited by the duration of follow-up for detecting potential benefits of midlife intensive BP control on late-life incidence of cognitive impairment.

## Conclusions

In this study, we did not observe an association of lower than usual BP targets with a reduction in the risk of cognitive decline, dementia, or MCI vs standard BP targets. The certainty of this evidence is low due to the limited follow-up period, the risk of bias of included trials, and the observed statistical heterogeneity. Hence, current available evidence does not justify the use of lower BP targets for the prevention of cognitive decline and dementia.

## Notes

Supplement. **eTable 1. **Database Search Strategy **eTable 2. **Additional Demographic Characteristics of Studies Included in Quantitative Analyses **eTable 3. **Subgroup Analysis for the Outcome of Cognitive Decline **eTable 4. **Subgroup Analysis for the Outcome of Probable Dementia **eTable 5. **Subgroup Analysis for the Outcome of Mild Cognitive Impairment **eTable 6. **Subgroup Analysis for the Outcome of Cerebrovascular Events **eTable 7. **Subgroup Analysis for the Outcome of Serious Adverse Events **eTable 8. **Subgroup Analysis for the Outcome of All-Cause Mortality **eFigure. **Sensitivity Analysis Using Fixed-Effect Model for the Incidence of Probable Dementia
