# Supplementary material for: Evaluation of Intensive vs Standard Blood Pressure Reduction and Association With Cognitive Decline and Dementia: A Systematic Review and Meta-analysis
Source: JAMA Netw Open. 2021 Nov 22;4(11):e2134553. doi: 10.1001/jamanetworkopen.2021.34553 (PMC8609411; doi:10.1001/jamanetworkopen.2021.34553)
Supplement: Supplement. — eTable 1. Database Search Strategy eTable 2. Additional Demographic Characteristics of Studies Included in Quantitative Analyses eTable 3. Subgroup Analysis for the Outcome of Cognitive Decline eTable 4. Subgroup Analysis for the Outcome of Probable Dementia eTable 5. Subgroup Analysis for the Outcome of Mild Cognitive Impairment eTable 6. Subgroup Analysis for the Outcome of Cerebrovascular Events eTable 7. Subgroup Analysis for the Outcome of Serious Adverse Events eTable 8. Subgroup Analysis for the Outcome of All-Cause Mortality eFigure. Sensitivity Analysis Using Fixed-Effect Model for the Incidence of Probable Dementia [file jamanetwopen-e2134553-s001.pdf]

Supplemental Online Content

Dallaire-Théroux C, Quesnel-Olivo MH, Brochu K, et al. Evaluation of intensive vs standard blood pressure reduction and association with cognitive decline and dementia: a systematic review and meta-analysis. *JAMA Netw Open.* 2021;4(11):e2134553. doi:10.1001/jamanetworkopen.2021.34553

eTable 1. Database Search Strategy

eTable 2. Additional Demographic Characteristics of Studies Included in Quantitative Analyses

eTable 3. Subgroup Analysis for the Outcome of Cognitive Decline

eTable 4. Subgroup Analysis for the Outcome of Probable Dementia

eTable 5. Subgroup Analysis for the Outcome of Mild Cognitive Impairment

eTable 6. Subgroup Analysis for the Outcome of Cerebrovascular Events

eTable 7. Subgroup Analysis for the Outcome of Serious Adverse Events

eTable 8. Subgroup Analysis for the Outcome of All-Cause Mortality

eFigure. Sensitivity Analysis Using Fixed-Effect Model for the Incidence of Probable Dementia

This supplemental material has been provided by the authors to give readers additional information about their work.

© 2021 Dallaire-Théroux C et al. *JAMA Network Open.*

eTable 1. Database Search Strategy

a. PubMed

Date of the search: 27-10-2020
Database limit: No database limit has been applied

Concepts	#	Search strategy	Results
Adult population (Controlled vocabulary)	1	Adult[MeSH]	7,274,737
Adult population (Free vocabulary)	2	Adult*[tiab] OR elder*[tiab] OR senior[tiab] OR aged[tiab] OR middle age[tiab] OR geriatri*[tiab] OR old person*[tiab] OR old patient[tiab] OR old people[tiab] OR older person*[tiab] OR older patient[tiab] OR older people[tiab]	2,059,980
Adult population (Combined)	3	#1 OR #2	8,241,625
Blood pressure reduction (Controlled vocabulary)	4	Antihypertensive Agents[MeSH] OR "Antihypertensive Agents" [Pharmacological Action] OR "Diuretics" [Pharmacological Action] OR "Diuretics"[Mesh] OR "Thiazides"[Mesh] OR "Calcium Channel Blockers"[Mesh] OR "Angiotensin-Converting Enzyme Inhibitors"[Mesh] OR "Angiotensin-Converting Enzyme Inhibitors" [Pharmacological Action] OR "Adrenergic beta-Antagonists" [Pharmacological Action]	391,258
Blood pressure reduction (Free vocabulary)	5	antihypertens*[tiab] OR anti-hypertens*[tiab] OR diuretic*[tiab] OR thiazide*[tiab] OR Calcium Channel Blocker*[tiab] OR Calcium Channel Antagonist*[tiab] OR calcium antagonist*[tiab] OR Angiotensin Converting Enzyme Inhibitors[tiab] OR ACE Inhibitors[tiab] OR angiotensin receptor blocker*[TIAB] OR beta Adrenergic Blockers[TIAB] OR beta-Adrenergic Antagonists[TIAB] OR "Adrenergic alpha-2 Receptor Agonists"[Mesh] OR beta-blocker*[TIAB] OR beta-Antagonist*[TIAB] OR alpha-2 agonist*[TIAB] OR alpha-1 blocker*[TIAB] OR Acebutolol[TIAB] OR Adrenomedullin[TIAB] OR AE0047[TIAB] OR alacepril[TIAB] OR Alprenolol[TIAB] OR ambrisentan[TIAB] OR Amlodipine[TIAB] OR amosulalol[TIAB] OR angiotensin I[TIAB] OR aprikalim[TIAB] OR Atenolol[TIAB] OR atrial natriuretic factor prohormone[TIAB] OR azepexole[TIAB] OR benazepril*[TIAB] OR bendazole[TIAB] OR Bendroflumethiazide[TIAB] OR benoxathian[TIAB] OR Bepridil[TIAB] OR berbamine[TIAB] OR Betaxolol[TIAB] OR Bethanidine[TIAB] OR bietaserpine[TIAB] OR bimakalim[TIAB] OR Bimatoprost[TIAB] OR Bisoprolol[TIAB] OR Bosentan[TIAB] OR BQ 22-708[TIAB] OR BQ 788[TIAB] OR Bretylium Tosylate[TIAB] OR Brimonidine Tartrate[TIAB] OR budralazine[TIAB] OR Bupranolol[TIAB] OR buthiazide[TIAB] OR cadralazine[TIAB] OR cafedrine[TIAB] OR candesartan[TIAB] OR	360,806

© 2021 Dallaire-Théroux C et al. *JAMA Network Open.*

		candoxatril[TIAB] OR Captopril[TIAB] OR Carteolol[TIAB] OR Carvedilol[TIAB]<br>OR Celiprolol[TIAB] OR ceronapril[TIAB] OR Chlorisondamine[TIAB]<br>OR Chlorothiazide[TIAB] OR Chlorthalidone[TIAB] OR cicletanine[TIAB] OR Cilazapril[TIAB] OR cilazaprilat[TIAB] OR clentiazem[TIAB] OR Clonidine[TIAB]<br>OR Cromakalim[TIAB] OR cyclo[TIAB] OR Cyclopenthiazide[TIAB] OR cyclothiazide[TIAB] OR dauricine[TIAB] OR Debrisoquin[TIAB] OR delapril[TIAB] OR diallyl disulfide[TIAB] OR Diazoxide[TIAB] OR Dihydralazine[TIAB] OR Dihydroalprenolol[TIAB] OR Diltiazem[TIAB] OR dorzolamide[TIAB] OR Doxazosin[TIAB] OR efonidipine[TIAB] OR Enalapril*[TIAB] OR epanolol[TIAB] OR Eplerenone[TIAB] OR Epoprostenol[TIAB] OR eprosartan[TIAB] OR essential 303 forte[TIAB] OR etozolin[TIAB] OR Felodipine[TIAB] OR Fenoldopam[TIAB] OR ferulic acid[TIAB] OR FK 409[TIAB] OR flesinoxan[TIAB] OR Fosinopril*[TIAB] OR grayanotoxin I[TIAB] OR Guanabenz[TIAB] OR guanadrel[TIAB] OR Guanethidine[TIAB] OR Guanfacine[TIAB] OR Hexamethonium[TIAB]<br>OR Hydralazine[TIAB] OR Hydrochlorothiazide[TIAB] OR Hydroflumethiazide[TIAB] OR imidapril[TIAB] OR Indapamide[TIAB] OR indenolol[TIAB] OR Indoramin[TIAB] OR indorenate[TIAB] OR Irbesartan[TIAB] OR isopropyl unoprostone[TIAB] OR Isradipine[TIAB]<br>OR Kallidin[TIAB] OR Ketanserin[TIAB] OR L 158809[TIAB] OR Labetalol[TIAB]<br>OR lacidipine[TIAB] OR Latanoprost[TIAB] OR lercanidipine[TIAB] OR libenzapril[TIAB] OR linsidomine[TIAB] OR Lisinopril[TIAB] OR lofexidine[TIAB] OR Losartan[TIAB] OR manidipine[TIAB] OR Mecamylamine[TIAB] OR medroxalol[TIAB] OR medullipin I[TIAB] OR Methyldopa[TIAB]<br>OR Metipranolol[TIAB] OR Metolazone[TIAB] OR Metoprolol[TIAB] OR Mibefradil[TIAB] OR Minoxidil[TIAB] OR moexipril[TIAB] OR monatepil[TIAB]<br>OR moxonidine[TIAB] OR Muzolimine[TIAB] OR Nadolol[TIAB] OR naftopidil[TIAB] OR Nebivolol[TIAB] OR Nicardipine[TIAB] OR Nicorandil[TIAB] OR niguldipine[TIAB] OR nilvadipine[TIAB] OR Nimodipine[TIAB] OR NIP 121[TIAB] OR nipradilol[TIAB] OR Nisoldipine[TIAB]<br>OR Nitrendipine[TIAB] OR Nitroprusside[TIAB] OR oleuropein[TIAB] OR olmesartan[TIAB] OR omapatrilat[TIAB] OR Oxprenolol[TIAB] OR Pargyline[TIAB] OR Pempidine[TIAB] OR Penbutolol[TIAB] OR Pentolinium Tartrate[TIAB] OR Perindopril*[TIAB] OR Phenoxybenzamine[TIAB] OR Phentolamine[TIAB] OR Pinacidil[TIAB] OR Pindolol[TIAB] OR Piperoxan[TIAB]<br>OR Polythiazide[TIAB] OR Prazosin[TIAB] OR Propranolol[TIAB] OR Protoveratrines[TIAB] OR	

© 2021 Dallaire-Théroux C et al. *JAMA Network Open.*

		Quinapril*[TIAB] OR Ramipril*[TIAB] OR remikiren[TIAB] OR rentiapril[TIAB] OR Reserpine[TIAB] OR Rilmenidine[TIAB] OR ryodipine[TIAB] OR saprisartan potassium[TIAB] OR scoparone [TIAB] OR selexipag[TIAB] OR sesamin[TIAB] OR spirapril[TIAB] OR talinolol[TIAB] OR Telmisartan[TIAB] OR temocapril hydrochloride[TIAB] OR Teprotide[TIAB] OR Terlipressin[TIAB] OR tetrahydropalmatine[TIAB] OR theodrenaline[TIAB] OR tibolone[TIAB] OR Ticrynafen[TIAB] OR Timolol[TIAB] OR tobanum[TIAB] OR tocopherylquinone[TIAB] OR Todralazine[TIAB] OR Tolazoline[TIAB] OR Torsemide[TIAB] OR trandolapril[TIAB] OR Travoprost[TIAB] OR treprostinil[TIAB] OR Trichlormethiazide[TIAB] OR trimazosin[TIAB] OR Trimethaphan[TIAB] OR urapidil[TIAB] OR Valsartan[TIAB] OR Veratrum Alkaloids[TIAB] OR Vincamine[TIAB] OR viprostol[TIAB] OR Xipamide[TIAB] OR Y 26763[TIAB] OR Y 27632[TIAB] OR zofenopril[TIAB]	
Blood pressure reduction (combined)	6	#4 OR #5	523,243
Cognition (Controlled vocabulary)	7	"Cognition"[Mesh:NoExp] OR "Cognitive Dysfunction"[Mesh:NoExp] OR "Cognition Disorders"[Mesh:NoExp] OR "Neurocognitive Disorders"[Mesh:NoExp] OR "Dementia"[Mesh] OR "Memory"[Mesh:NoExp]	373,550
Cognition (Free vocabulary)	8	Cogn*[TIAB] or dement*[tiab] OR neurocogn*[TIAB] OR alzheimer*[tiab] OR (mental[TIAB] AND (Disorders[TIAB] OR decline[TIAB] OR impairment[TIAB] OR Deterioration[TIAB] OR syndromes[TIAB] OR Dysfunction[TIAB]))	687,538
Cognition (combined)	9	#7 OR #8	804,666
Randomized controlled clinical trials search filter	10	((randomized controlled trial[pt]) OR (controlled clinical trial[pt]) OR (randomized[tiab] OR randomised[tiab]) OR (placebo[tiab]) OR (drug therapy[sh]) OR (randomly[tiab]) OR (trial[tiab]) OR (groups[tiab]))	4,910,325
Combination of concepts	11	#3 AND 6 AND #9 AND #10	2,109
Human studies only	12	#11 NOT (animals[MeSH] NOT humans[MeSH])	2,006

© 2021 Dallaire-Théroux C et al. *JAMA Network Open.*

b. Embase (Embase.com)

Date of the search: 27-10-2020
Database limit: No database limit has been applied

Concepts	#	Search strategy	Results
Adult population (Controlled vocabulary)	1	'adult'/exp OR 'aged'/exp	8,947,301
Adult population (Free vocabulary)	2	Adult$:ab,ti OR (old* NEAR/2 (people OR person$ OR adult$ OR patient)):ab,ti OR elder*:ab,ti OR aged:ab,ti OR senior$:ab,ti OR geriatri*:ti,ab OR "middle age":ab,ti	2,754,485
Adult population (Combined)	3	#1 OR #2	9,980,405
Blood pressure reduction (Controlled vocabulary)	4	'antihypertensive agent'/exp	758,646
Blood pressure reduction (Free vocabulary)	5	antihypertens*:ti,ab OR "anti-hypertens*":ti,ab OR diuretic$:ti,ab OR thiazide$:ti,ab OR (calcium NEAR/2 (antagonist$ OR Blocker$)):ti,ab OR “angiotensin converting enzyme inhibitors”:ti,ab OR "ACE Inhibitor$":ti,ab OR "angiotensin receptor blocker$":ti,ab OR Acebutolol:ti,ab OR Adrenomedullin:ti,ab OR AE0047:ti,ab OR alacepril:ti,ab OR Alprenolol:ti,ab OR ambrisentan:ti,ab OR Amlodipine:ti,ab OR amosulalol:ti,ab OR “angiotensin I”:ti,ab OR aprikalim:ti,ab OR Atenolol:ti,ab OR "atrial natriuretic factor prohormone":ti,ab OR azepexole:ti,ab OR benazepril*:ti,ab OR bendazole:ti,ab OR Bendroflumethiazide:ti,ab OR benoxathian:ti,ab OR Bepridil:ti,ab OR berbamine:ti,ab OR Betaxolol:ti,ab OR Bethanidine:ti,ab OR bietaserpine:ti,ab OR bimakalim:ti,ab OR Bimatoprost:ti,ab OR Bisoprolol:ti,ab OR Bosentan:ti,ab OR "BQ 22-708":ti,ab OR "BQ 788":ti,ab OR "Bretylium Tosylate":ti,ab OR "Brimonidine Tartrate":ti,ab OR budralazine:ti,ab OR Bupranolol:ti,ab OR buthiazide:ti,ab OR cadralazine:ti,ab OR cafedrine:ti,ab OR candesartan:ti,ab OR candoxatril:ti,ab OR Captopril:ti,ab OR Carteolol:ti,ab OR Carvedilol:ti,ab OR Celiprolol:ti,ab OR ceronapril:ti,ab OR Chlorisondamine:ti,ab OR Chlorothiazide:ti,ab OR Chlorthalidone:ti,ab OR cicletanine:ti,ab OR Cilazapril:ti,ab OR cilazaprilat:ti,ab OR clentiazem:ti,ab OR Clonidine:ti,ab OR Cromakalim:ti,ab OR cyclo:ti,ab OR Cyclopenthiazide:ti,ab OR cyclothiazide:ti,ab OR dauricine:ti,ab OR Debrisoquin:ti,ab OR delapril:ti,ab OR "diallyl disulfide":ti,ab OR Diazoxide:ti,ab OR Dihydralazine:ti,ab OR Dihydroalprenolol:ti,ab OR Diltiazem:ti,ab OR dorzolamide:ti,ab OR Doxazosin:ti,ab OR efonidipine:ti,ab OR Enalapril*:ti,ab OR epanolol:ti,ab OR Eplerenone:ti,ab OR Epoprostenol:ti,ab OR eprosartan:ti,ab OR "essential 303 forte":ti,ab OR etozolin:ti,ab OR Felodipine:ti,ab OR Fenoldopam:ti,ab OR "ferulic acid":ti,ab OR "FK 409":ti,ab OR flesinoxan:ti,ab OR Fosinopril*:ti,ab OR grayanotoxin I:ti,ab OR Guanabenz:ti,ab OR guanadrel:ti,ab OR Guanethidine:ti,ab OR Guanfacine:ti,ab OR	246,876

© 2021 Dallaire-Théroux C et al. *JAMA Network Open.*

		Hexamethonium:ti,ab OR Hydralazine:ti,ab OR Hydrochlorothiazide:ti,ab OR Hydroflumethiazide:ti,ab OR imidapril:ti,ab OR Indapamide:ti,ab OR indenolol:ti,ab OR Indoramin:ti,ab OR indorenate:ti,ab OR Irbesartan:ti,ab OR "isopropyl unoprostone":ti,ab OR Isradipine:ti,ab OR Kallidin:ti,ab OR Ketanserin:ti,ab OR "L 158809":ti,ab OR Labetalol:ti,ab OR lacidipine:ti,ab OR Latanoprost:ti,ab OR lercanidipine:ti,ab OR libenzapril:ti,ab OR linsidomine:ti,ab OR Lisinopril:ti,ab OR lofexidine:ti,ab OR Losartan:ti,ab OR manidipine:ti,ab OR Mecamylamine:ti,ab OR medroxalol:ti,ab OR "medullipin I":ti,ab OR Methyldopa:ti,ab OR Metipranolol:ti,ab OR Metolazone:ti,ab OR Metoprolol:ti,ab OR Mibefradil:ti,ab OR Minoxidil:ti,ab OR moexipril:ti,ab OR monatepil:ti,ab OR moxonidine:ti,ab OR Muzolimine:ti,ab OR Nadolol:ti,ab OR naftopidil:ti,ab OR Nebivolol:ti,ab OR Nicardipine:ti,ab OR Nicorandil:ti,ab OR niguldipine:ti,ab OR nilvadipine:ti,ab OR Nimodipine:ti,ab OR "NIP 121":ti,ab OR nipradilol:ti,ab OR Nisoldipine:ti,ab OR Nitrendipine:ti,ab OR Nitroprusside:ti,ab OR oleuropein:ti,ab OR olmesartan:ti,ab OR omapatrilat:ti,ab OR Oxprenolol:ti,ab OR Pargyline:ti,ab OR Pempidine:ti,ab OR Penbutolol:ti,ab OR "Pentolinium Tartrate":ti,ab OR Perindopril*:ti,ab OR Phenoxybenzamine:ti,ab OR Phentolamine:ti,ab OR Pinacidil:ti,ab OR Pindolol:ti,ab OR Piperoxan:ti,ab OR Polythiazide:ti,ab OR Prazosin:ti,ab OR Propranolol:ti,ab OR Protoveratrines:ti,ab OR Quinapril*:ti,ab OR Ramipril*:ti,ab OR remikiren:ti,ab OR rentiapril:ti,ab OR Reserpine:ti,ab OR Rilmenidine:ti,ab OR ryodipine:ti,ab OR "saprisartan potassium":ti,ab OR scoparone:ti,ab OR selexipag:ti,ab OR sesamin:ti,ab OR spirapril:ti,ab OR talinolol:ti,ab OR Telmisartan:ti,ab OR "temocapril hydrochloride":ti,ab OR Teprotide:ti,ab OR Terlipressin:ti,ab OR tetrahydropalmatine:ti,ab OR theodrenaline:ti,ab OR tibolone:ti,ab OR Ticrynafen:ti,ab OR Timolol:ti,ab OR tobanum:ti,ab OR tocopherylquinone:ti,ab OR Todralazine:ti,ab OR Tolazoline:ti,ab OR Torsemide:ti,ab OR trandolapril:ti,ab OR Travoprost:ti,ab OR treprostinil:ti,ab OR Trichlormethiazide:ti,ab OR trimazosin:ti,ab OR Trimethaphan:ti,ab OR urapidil:ti,ab OR Valsartan:ti,ab OR "Veratrum Alkaloids":ti,ab OR Vincamine:ti,ab OR viprostol:ti,ab OR Xipamide:ti,ab OR "Y 26763":ti,ab OR "Y 27632":ti,ab OR zofenopril:ti,ab	
Blood pressure reduction (combined)	6	#4 OR #5	815,353
Cognition (Controlled vocabulary)	7	'cognition'/de OR 'cognitive defect'/de OR 'dementia'/exp OR 'mild cognitive impairment'/de OR 'disorders of higher cerebral function'/de OR 'memory'/de	778,993
Cognition (Free vocabulary)	8	Cogn*:ab,ti or dement*:ab,ti OR neurocogn*:ab,ti OR alzheimer*:ab,ti OR (mental NEAR/2 (Disorders OR decline OR impairment OR Deterioration OR syndromes OR Dysfunction)):ab,ti	861,245
Cognition	9	#7 OR #8	1,123,687

© 2021 Dallaire-Théroux C et al. *JAMA Network Open.*

(combined)			
Randomized controlled clinical trials search filter	10	'randomized controlled trial'/de OR 'controlled clinical trial'/de OR randomized:ab,ti OR randomised:ab,ti OR placebo:ab,ti OR randomly:ab,ti OR trial:ab,ti OR groups:ab,ti OR 'drug therapy':lnk	7,597,657
Combination of concepts	11	#3 AND 6 AND #9 AND #10	5,592
Human studies only	12	#11 NOT ('animal'/exp NOT 'human'/exp)	5,385

© 2021 Dallaire-Théroux C et al. *JAMA Network Open.*

c. Cochrane Library

Date of the search: 27-10-2020
Database limit: limit results to trials only

Concepts	#	Search strategy	Results
Adult population (Controlled vocabulary)	1	[mh Adult]	458209
Adult population (Free vocabulary)	2	Adult?:ab,ti OR (old* NEAR/2 (people OR person? OR adult? OR patient)):ab,ti OR elder*:ab,ti OR aged:ab,ti OR senior$:ab,ti OR geriatri*:ti,ab OR "middle age":ab,ti	261941
Adult population (Combined)	3	#1 OR #2	643083
Blood pressure reduction (Controlled vocabulary)	4	[mh "Antihypertensive Agents"] OR [mh Diuretics] OR [mh Thiazides] OR [mh "Calcium Channel Blockers"] OR [mh "Angiotensin-Converting Enzyme Inhibitors"] OR [mh "Adrenergic beta-Antagonists"] OR [mh "Adrenergic alpha-2 Receptor Agonists"]	19674
Blood pressure reduction (Free vocabulary)	5	antihypertens*:ti,ab OR "anti-hypertens*":ti,ab OR diuretic$:ti,ab OR thiazide$:ti,ab OR (calcium NEAR/2 (antagonist$ OR Blocker$)):ti,ab OR “angiotensin converting enzyme inhibitors”:ti,ab OR "ACE Inhibitor$":ti,ab OR "angiotensin receptor blocker$":ti,ab OR Acebutolol:ti,ab OR Adrenomedullin:ti,ab OR AE0047:ti,ab OR alacepril:ti,ab OR Alprenolol:ti,ab OR ambrisentan:ti,ab OR Amlodipine:ti,ab OR amosulalol:ti,ab OR “angiotensin I”:ti,ab OR aprikalim:ti,ab OR Atenolol:ti,ab OR "atrial natriuretic factor prohormone":ti,ab OR azepexole:ti,ab OR benazepril*:ti,ab OR bendazole:ti,ab OR Bendroflumethiazide:ti,ab OR benoxathian:ti,ab OR Bepridil:ti,ab OR berbamine:ti,ab OR Betaxolol:ti,ab OR Bethanidine:ti,ab OR bietaserpine:ti,ab OR bimakalim:ti,ab OR Bimatoprost:ti,ab OR Bisoprolol:ti,ab OR Bosentan:ti,ab OR "BQ 22-708":ti,ab OR "BQ 788":ti,ab OR "Bretylium Tosylate":ti,ab OR "Brimonidine Tartrate":ti,ab OR budralazine:ti,ab OR Bupranolol:ti,ab OR buthiazide:ti,ab OR cadralazine:ti,ab OR cafedrine:ti,ab OR candesartan:ti,ab OR candoxatril:ti,ab OR Captopril:ti,ab OR Carteolol:ti,ab OR Carvedilol:ti,ab OR Celiprolol:ti,ab OR ceronapril:ti,ab OR Chlorisondamine:ti,ab OR Chlorothiazide:ti,ab OR Chlorthalidone:ti,ab OR cicletanine:ti,ab OR Cilazapril:ti,ab OR cilazaprilat:ti,ab OR clentiazem:ti,ab OR Clonidine:ti,ab OR Cromakalim:ti,ab OR cyclo:ti,ab OR Cyclopenthiazide:ti,ab OR cyclothiazide:ti,ab OR dauricine:ti,ab OR Debrisoquin:ti,ab OR delapril:ti,ab OR "diallyl disulfide":ti,ab OR Diazoxide:ti,ab OR Dihydralazine:ti,ab OR Dihydroalprenolol:ti,ab OR Diltiazem:ti,ab OR dorzolamide:ti,ab OR Doxazosin:ti,ab OR efonidipine:ti,ab OR Enalapril*:ti,ab OR epanolol:ti,ab OR Eplerenone:ti,ab OR Epoprostenol:ti,ab OR eprosartan:ti,ab OR "essential 303 forte":ti,ab OR etozolin:ti,ab OR Felodipine:ti,ab OR Fenoldopam:ti,ab OR "ferulic acid":ti,ab OR "FK 409":ti,ab OR flesinoxan:ti,ab OR Fosinopril*:ti,ab OR grayanotoxin I:ti,ab OR Guanabenz:ti,ab OR guanadrel:ti,ab OR	57307

© 2021 Dallaire-Théroux C et al. *JAMA Network Open.*

		Guanethidine:ti,ab OR Guanfacine:ti,ab OR Hexamethonium:ti,ab OR Hydralazine:ti,ab OR Hydrochlorothiazide:ti,ab OR Hydroflumethiazide:ti,ab OR imidapril:ti,ab OR Indapamide:ti,ab OR indenolol:ti,ab OR Indoramin:ti,ab OR indorenate:ti,ab OR Irbesartan:ti,ab OR "isopropyl unoprostone":ti,ab OR Isradipine:ti,ab OR Kallidin:ti,ab OR Ketanserin:ti,ab OR "L 158809":ti,ab OR Labetalol:ti,ab OR lacidipine:ti,ab OR Latanoprost:ti,ab OR lercanidipine:ti,ab OR libenzapril:ti,ab OR linsidomine:ti,ab OR Lisinopril:ti,ab OR lofexidine:ti,ab OR Losartan:ti,ab OR manidipine:ti,ab OR Mecamylamine:ti,ab OR medroxalol:ti,ab OR "medullipin I":ti,ab OR Methyldopa:ti,ab OR Metipranolol:ti,ab OR Metolazone:ti,ab OR Metoprolol:ti,ab OR Mibefradil:ti,ab OR Minoxidil:ti,ab OR moexipril:ti,ab OR monatepil:ti,ab OR moxonidine:ti,ab OR Muzolimine:ti,ab OR Nadolol:ti,ab OR naftopidil:ti,ab OR Nebivolol:ti,ab OR Nicardipine:ti,ab OR Nicorandil:ti,ab OR niguldipine:ti,ab OR nilvadipine:ti,ab OR Nimodipine:ti,ab OR "NIP 121":ti,ab OR nipradilol:ti,ab OR Nisoldipine:ti,ab OR Nitrendipine:ti,ab OR Nitroprusside:ti,ab OR oleuropein:ti,ab OR olmesartan:ti,ab OR omapatrilat:ti,ab OR Oxprenolol:ti,ab OR Pargyline:ti,ab OR Pempidine:ti,ab OR Penbutolol:ti,ab OR "Pentolinium Tartrate":ti,ab OR Perindopril*:ti,ab OR Phenoxybenzamine:ti,ab OR Phentolamine:ti,ab OR Pinacidil:ti,ab OR Pindolol:ti,ab OR Piperoxan:ti,ab OR Polythiazide:ti,ab OR Prazosin:ti,ab OR Propranolol:ti,ab OR Protoveratrines:ti,ab OR Quinapril*:ti,ab OR Ramipril*:ti,ab OR remikiren:ti,ab OR rentiapril:ti,ab OR Reserpine:ti,ab OR Rilmenidine:ti,ab OR ryodipine:ti,ab OR "saprisartan potassium":ti,ab OR scoparone:ti,ab OR selexipag:ti,ab OR sesamin:ti,ab OR spirapril:ti,ab OR talinolol:ti,ab OR Telmisartan:ti,ab OR "temocapril hydrochloride":ti,ab OR Teprotide:ti,ab OR Terlipressin:ti,ab OR tetrahydropalmatine:ti,ab OR theodrenaline:ti,ab OR tibolone:ti,ab OR Ticrynafen:ti,ab OR Timolol:ti,ab OR tobanum:ti,ab OR tocopherylquinone:ti,ab OR Todralazine:ti,ab OR Tolazoline:ti,ab OR Torsemide:ti,ab OR trandolapril:ti,ab OR Travoprost:ti,ab OR treprostinil:ti,ab OR Trichlormethiazide:ti,ab OR trimazosin:ti,ab OR Trimethaphan:ti,ab OR urapidil:ti,ab OR Valsartan:ti,ab OR "Veratrum Alkaloids":ti,ab OR Vincamine:ti,ab OR viprostol:ti,ab OR Xipamide:ti,ab OR "Y 26763":ti,ab OR "Y 27632":ti,ab OR zofenopril:ti,ab	
Blood pressure reduction (combined)	6	#4 OR #5	62225
Cognition (Controlled vocabulary)	7	[mh ^"Cognition"] OR [mh ^"Cognitive Dysfunction"] OR [mh ^"Cognition Disorders"] OR [mh ^"Neurocognitive Disorders"] OR [mh "Dementia"] OR [mh ^"Memory"]	18154
Cognition (Free vocabulary)	8	Cogn*:ab,ti or dement*:ab,ti OR neurocogn*:ab,ti OR alzheimer*:ab,ti OR (mental NEAR/2 (Disorders OR decline OR impairment OR Deterioration OR syndromes OR Dysfunction)):ab,ti	88420
Cognition	9	#7 OR #8	

© 2021 Dallaire-Théroux C et al. *JAMA Network Open.*

(combined)			
Combination of concepts	10	#3 AND #6 AND #9	736
Trials only	11	n/a	726

© 2021 Dallaire-Théroux C et al. *JAMA Network Open.*

d. PsycInfo (OVID)

Date of the search: 27-10-2020
Database limit: No database limit has been applied

Concepts	#	Search strategy	Results
Adult population (Controlled vocabulary)	1	300.ag	1933385
Adult population (Free vocabulary)	2	Adult?.ab,ti OR (old* adj2 (people OR person? OR adult? OR patient)).ab,ti OR elder*.ab,ti OR aged.ab,ti OR senior?.ab,ti OR geriatri*.ti,ab OR "middle age".ab,ti	706114
Adult population (Combined)	3	1 OR 2	2178411
Blood pressure reduction (Controlled vocabulary)	4	exp antihypertensive drugs/ OR exp diuretics/ OR channel blockers/ OR (Angiotensin/ AND Enzyme Inhibitors/) OR Adrenergic Receptors/	11029
Blood pressure reduction (Free vocabulary)	5	antihypertens*.ti,ab OR "anti-hypertens*".ti,ab OR diuretic?.ti,ab OR thiazide?.ti,ab OR (calcium adj2 (antagonist? OR Blocker?)).ti,ab OR "angiotensin converting enzyme inhibitors".ti,ab OR "ACE Inhibitor?".ti,ab OR "angiotensin receptor blocker?".ti,ab OR Acebutolol.ti,ab OR Adrenomedullin.ti,ab OR AE0047.ti,ab OR alacepril.ti,ab OR Alprenolol.ti,ab OR ambrisentan.ti,ab OR Amlodipine.ti,ab OR amosulalol.ti,ab OR "angiotensin I".ti,ab OR aprikalim.ti,ab OR Atenolol.ti,ab OR "atrial natriuretic factor prohormone".ti,ab OR azepexole.ti,ab OR benazepril*.ti,ab OR bendazole.ti,ab OR Bendroflumethiazide.ti,ab OR benoxathian.ti,ab OR Bepridil.ti,ab OR berbamine.ti,ab OR Betaxolol.ti,ab OR Bethanidine.ti,ab OR bietaserpine.ti,ab OR bimakalim.ti,ab OR Bimatoprost.ti,ab OR Bisoprolol.ti,ab OR Bosentan.ti,ab OR "BQ 22-708".ti,ab OR "BQ 788".ti,ab OR "Bretylium Tosylate".ti,ab OR "Brimonidine Tartrate".ti,ab OR budralazine.ti,ab OR Bupranolol.ti,ab OR buthiazide.ti,ab OR cadralazine.ti,ab OR cafedrine.ti,ab OR candesrtan.ti,ab OR candoxatril.ti,ab OR Captopril.ti,ab OR Carteolol.ti,ab OR Carvedilol.ti,ab OR Celiprolol.ti,ab OR ceronapril.ti,ab OR Chlorisondamine.ti,ab OR Chlorothiazide.ti,ab OR Chlorthalidone.ti,ab OR cicletanine.ti,ab OR Cilazapril.ti,ab OR cilazaprilat.ti,ab OR clentiazem.ti,ab OR Clonidine.ti,ab OR Cromakalim.ti,ab OR cyclo.ti,ab OR Cyclopenthiazide.ti,ab OR cyclothiazide.ti,ab OR dauricine.ti,ab OR Debrisoquin.ti,ab OR delapril.ti,ab OR "diallyl disulfide".ti,ab OR Diazoxide.ti,ab OR Dihydralazine.ti,ab OR Dihydroalprenolol.ti,ab OR Diltiazem.ti,ab OR dorzolamide.ti,ab OR Doxazosin.ti,ab OR efonidipine.ti,ab OR Enalapril*.ti,ab OR epanolol.ti,ab OR Eplerenone.ti,ab OR Epoprostenol.ti,ab OR eprosartan.ti,ab OR "essential 303 forte".ti,ab OR etozolin.ti,ab OR Felodipine.ti,ab OR Fenoldopam.ti,ab OR "ferulic acid".ti,ab OR "FK 409".ti,ab OR flesinoxan.ti,ab OR Fosinopril*.ti,ab OR grayanotoxin I.ti,ab OR Guanabenz.ti,ab OR guanadrel.ti,ab OR Guanethidine.ti,ab OR Guanfacine.ti,ab OR	14975

© 2021 Dallaire-Théroux C et al. *JAMA Network Open.*

		Hexamethonium.ti,ab OR Hydralazine.ti,ab OR Hydrochlorothiazide.ti,ab OR Hydroflumethiazide.ti,ab OR imidapril.ti,ab OR Indapamide.ti,ab OR indenolol.ti,ab OR Indoramin.ti,ab OR indorenate.ti,ab OR Irbesartan.ti,ab OR "isopropyl unoprostone".ti,ab OR Isradipine.ti,ab OR Kallidin.ti,ab OR Ketanserin.ti,ab OR "L 158809".ti,ab OR Labetalol.ti,ab OR lacidipine.ti,ab OR Latanoprost.ti,ab OR lercanidipine.ti,ab OR libenzapril.ti,ab OR linsidomine.ti,ab OR Lisinopril.ti,ab OR lofexidine.ti,ab OR Losartan.ti,ab OR manidipine.ti,ab OR Mecamylamine.ti,ab OR medroxalol.ti,ab OR "medullipin I".ti,ab OR Methyldopa.ti,ab OR Metipranolol.ti,ab OR Metolazone.ti,ab OR Metoprolol.ti,ab OR Mibefradil.ti,ab OR Minoxidil.ti,ab OR moexipril.ti,ab OR monatepil.ti,ab OR moxonidine.ti,ab OR Muzolimine.ti,ab OR Nadolol.ti,ab OR naftopidil.ti,ab OR Nebivolol.ti,ab OR Nicardipine.ti,ab OR Nicorandil.ti,ab OR niguldipine.ti,ab OR nilvadipine.ti,ab OR Nimodipine.ti,ab OR "NIP 121".ti,ab OR nipradilol.ti,ab OR Nisoldipine.ti,ab OR Nitrendipine.ti,ab OR Nitroprusside.ti,ab OR oleuropein.ti,ab OR olmesartan.ti,ab OR omapatrilat.ti,ab OR Oxprenolol.ti,ab OR Pargyline.ti,ab OR Pempidine.ti,ab OR Penbutolol.ti,ab OR "Pentolinium Tartrate".ti,ab OR Perindopril*.ti,ab OR Phenoxybenzamine.ti,ab OR Phentolamine.ti,ab OR Pinacidil.ti,ab OR Pindolol.ti,ab OR Piperoxan.ti,ab OR Polythiazide.ti,ab OR Prazosin.ti,ab OR Propranolol.ti,ab OR Protoveratrines.ti,ab OR Quinapril*.ti,ab OR Ramipril*.ti,ab OR remikiren.ti,ab OR rentiapril.ti,ab OR Reserpine.ti,ab OR Rilmenidine.ti,ab OR ryodipine.ti,ab OR "saprisartan potassium".ti,ab OR scoparone.ti,ab OR selexipag.ti,ab OR sesamin.ti,ab OR spirapril.ti,ab OR talinolol.ti,ab OR Telmisartan.ti,ab OR "temocapril hydrochloride".ti,ab OR Teprotide.ti,ab OR Terlipressin.ti,ab OR tetrahydropalmatine.ti,ab OR theodrenaline.ti,ab OR tibolone.ti,ab OR Ticrynafen.ti,ab OR Timolol.ti,ab OR tobanum.ti,ab OR tocopherylquinone.ti,ab OR Todralazine.ti,ab OR Tolazoline.ti,ab OR Torsemide.ti,ab OR trandolapril.ti,ab OR Travoprost.ti,ab OR treprostinil.ti,ab OR Trichlormethiazide.ti,ab OR trimazosin.ti,ab OR Trimethaphan.ti,ab OR urapidil.ti,ab OR Valsartan.ti,ab OR "Veratrum Alkaloids".ti,ab OR Vincamine.ti,ab OR viprostol.ti,ab OR Xipamide.ti,ab OR "Y 26763".ti,ab OR "Y 27632".ti,ab OR zofenopril.ti,ab	
Blood pressure reduction (combined)	6	4 OR 5	21892
Cognition (Controlled vocabulary)	7	Cognition/ OR Cognitive Impairment/ OR Mild Cognitive Impairment/ OR exp dementia/ OR Memory/ OR Neurocognitive Disorders/ OR Alzheimer's Disease/	196056
Cognition (Free vocabulary)	8	Cogn*.ti,ab or dement*.ti,ab OR neurocogn*.ti,ab OR alzheimer*.ti,ab OR (mental adj2 (Disorders OR decline OR impairment OR Deterioration OR syndromes OR Dysfunction)).ti,ab	576586
Cognition	9	7 OR 8	634280

© 2021 Dallaire-Théroux C et al. *JAMA Network Open.*

(combined)			
Randomized controlled clinical trials search filter	10	Randomized Controlled Trial/ OR Drug Therapy/ OR randomized.ab,ti OR randomised.ab,ti OR placebo.ab,ti OR randomly.ab,ti OR trial.ab,ti OR groups.ab,ti	774771
Combination of concepts	11	3 AND 6 AND 9 AND 10	767

© 2021 Dallaire-Théroux C et al. *JAMA Network Open.*

e. CINAHL
Date of the search: 27-10-2020
Database limit: No database limit has been applied

Concepts	#	Search strategy	Results
Adult population (Controlled vocabulary)	1	MH ("Adult+" OR "Aged+" OR "Aged, 80 and Over+")	1,834,613
Adult population (Free vocabulary)	2	TI (Adult? OR (old* N2 (people OR person? OR adult? OR patient)) OR elder* OR aged OR senior? OR geriatri* OR "middle age") OR AB (Adult? OR (old* N2 (people OR person? OR adult? OR patient)) OR elder* OR aged OR senior? OR geriatri* OR "middle age")	600,863
Adult population (Combined)	3	S1 OR S2	2,061,707
Blood pressure reduction (Controlled vocabulary)	4	MH ("Antihypertensive Agents+" OR "Diuretics+" OR "Diuretics, Thiazide" OR "Calcium Channel Blockers" OR "Calcium Channel Agonists" OR "Angiotensin-Converting Enzyme Inhibitors" OR "Adrenergic Beta-Antagonists+" OR "Receptors, Adrenergic, Alpha")	47,136
Blood pressure reduction (Free vocabulary)	5	TI (antihypertens* OR "anti-hypertens*" OR diuretic? OR thiazide? OR (calcium adj2 (antagonist? OR Blocker?)) OR "angiotensin converting enzyme inhibitors" OR "ACE Inhibitor?" OR "angiotensin receptor blocker?" OR Acebutolol OR Adrenomedullin OR AE0047 OR alacepril OR Alprenolol OR ambrisentan OR Amlodipine OR amosulalol OR "angiotensin I" OR aprikalim OR Atenolol OR "atrial natriuretic factor prohormone" OR azepexole OR benazepril* OR bendazole OR Bendroflumethiazide OR benoxathian OR Bepridil OR berbamine OR Betaxolol OR Bethanidine OR bietaserpine OR bimakalim OR Bimatoprost OR Bisoprolol OR Bosentan OR "BQ 22-708" OR "BQ 788" OR "Bretylium Tosylate" OR "Brimonidine Tartrate" OR budralazine OR Bupranolol OR buthiazide OR cadralazine OR cafedrine OR candesrtan OR candoxatril OR Captopril OR Carteolol OR Carvedilol OR Celiprolol OR ceronapril OR Chlorisondamine OR Chlorothiazide OR Chlorthalidone OR cicletanine OR Cilazapril OR cilazaprilat OR clentiazem OR Clonidine OR Cromakalim OR cyclo OR Cyclopenthiazide OR cyclothiazide OR dauricine OR Debrisoquin OR delapril OR "diallyl disulfide" OR Diazoxide OR Dihydralazine OR Dihydroalprenolol OR Diltiazem OR dorzolamide OR Doxazosin OR efonidipine OR Enalapril* OR epanolol OR Eplerenone OR Epoprostenol OR eprosartan OR "essential 303 forte" OR etozolin OR Felodipine OR Fenoldopam OR "ferulic acid" OR "FK 409" OR flesinoxan OR Fosinopril* OR grayanotoxin I OR Guanabenz OR guanadrel OR Guanethidine OR Guanfacine OR Hexamethonium OR Hydralazine OR Hydrochlorothiazide OR Hydroflumethiazide OR imidapril OR Indapamide OR indenolol OR Indoramin OR indorenate OR Irbesartan OR "isopropyl unoprostone" OR Isradipine OR Kallidin OR	37,696

© 2021 Dallaire-Théroux C et al. *JAMA Network Open.*

		Ketanserin OR "L 158809" OR Labetalol OR lacidipine OR Latanoprost OR lercanidipine OR libenzapril OR linsidomine OR Lisinopril OR lofexidine OR Losartan OR manidipine OR Mecamylamine OR medroxalol OR "medullipin I" OR Methyldopa OR Metipranolol OR Metolazone OR Metoprolol OR Mibefradil OR Minoxidil OR moexipril OR monatepil OR moxonidine OR Muzolimine OR Nadolol OR naftopidil OR Nebivolol OR Nicardipine OR Nicorandil OR niguldipine OR nilvadipine OR Nimodipine OR "NIP 121" OR nipradilol OR Nisoldipine OR Nitrendipine OR Nitroprusside OR oleuropein OR olmesartan OR omapatrilat OR Oxprenolol OR Pargyline OR Pempidine OR Penbutolol OR "Pentolinium Tartrate" OR Perindopril* OR Phenoxybenzamine OR Phentolamine OR Pinacidil OR Pindolol OR Piperoxan OR Polythiazide OR Prazosin OR Propranolol OR Protoveratrines OR Quinapril* OR Ramipril* OR remikiren OR rentiapril OR Reserpine OR Rilmenidine OR ryodipine OR "saprisartan potassium" OR scoparone OR selexipag OR sesamin OR spirapril OR talinolol OR Telmisartan OR "temocapril hydrochloride" OR Teprotide OR Terlipressin OR tetrahydropalmatine OR theodrenaline OR tibolone OR Ticrynafen OR Timolol OR tobanum OR tocopherylquinone OR Todralazine OR Tolazoline OR Torsemide OR trandolapril OR Travoprost OR treprostinil OR Trichlormethiazide OR trimazosin OR Trimethaphan OR urapidil OR Valsartan OR "Veratrum Alkaloids" OR Vincamine OR viprostol OR Xipamide OR "Y 26763" OR "Y 27632" OR zofenopril) OR AB (antihypertens* OR "anti-hypertens*" OR diuretic? OR thiazide? OR (calcium adj2 (antagonist? OR Blocker?)) OR "angiotensin converting enzyme inhibitors" OR "ACE Inhibitor?" OR "angiotensin receptor blocker?" OR Acebutolol OR Adrenomedullin OR AE0047 OR alacepril OR Alprenolol OR ambrisentan OR Amlodipine OR amosulalol OR "angiotensin I" OR aprikalim OR Atenolol OR "atrial natriuretic factor prohormone" OR azepexole OR benazepril* OR bendazole OR Bendroflumethiazide OR benoxathian OR Bepridil OR berbamine OR Betaxolol OR Bethanidine OR bietaserpine OR bimakalim OR Bimatoprost OR Bisoprolol OR Bosentan OR "BQ 22-708" OR "BQ 788" OR "Bretylium Tosylate" OR "Brimonidine Tartrate" OR budralazine OR Bupranolol OR buthiazide OR cadralazine OR cafedrine OR candesrtan OR candoxatril OR Captopril OR Carteolol OR Carvedilol OR Celiprolol OR ceronapril OR Chlorisondamine OR Chlorothiazide OR Chlorthalidone OR cicletanine OR Cilazapril OR cilazaprilat OR clentiazem OR Clonidine OR Cromakalim OR cyclo OR Cyclopenthiazide OR cyclothiazide OR dauricine OR Debrisoquin OR delapril OR "diallyl disulfide" OR Diazoxide OR Dihydralazine OR Dihydroalprenolol OR Diltiazem OR dorzolamide OR Doxazosin OR efonidipine OR Enalapril* OR epanolol OR Eplerenone OR Epoprostenol OR eprosartan OR "essential 303 forte" OR etozolin OR Felodipine OR Fenoldopam OR "ferulic acid" OR "FK 409" OR flesinoxan	

© 2021 Dallaire-Théroux C et al. *JAMA Network Open.*

		OR Fosinopril* OR grayanotoxin I OR Guanabenz OR guanadrel OR Guanethidine OR Guanfacine OR Hexamethonium OR Hydralazine OR Hydrochlorothiazide OR Hydroflumethiazide OR imidapril OR Indapamide OR indenolol OR Indoramin OR indorenate OR Irbesartan OR "isopropyl unoprostone" OR Isradipine OR Kallidin OR Ketanserin OR "L 158809" OR Labetalol OR lacidipine OR Latanoprost OR lercanidipine OR libenzapril OR linsidomine OR Lisinopril OR lofexidine OR Losartan OR manidipine OR Mecamylamine OR medroxalol OR "medullipin I" OR Methyldopa OR Metipranolol OR Metolazone OR Metoprolol OR Mibefradil OR Minoxidil OR moexipril OR monatepil OR moxonidine OR Muzolimine OR Nadolol OR naftopidil OR Nebivolol OR Nicardipine OR Nicorandil OR niguldipine OR nilvadipine OR Nimodipine OR "NIP 121" OR nipradilol OR Nisoldipine OR Nitrendipine OR Nitroprusside OR oleuropein OR olmesartan OR omapatrilat OR Oxprenolol OR Pargyline OR Pempidine OR Penbutolol OR "Pentolinium Tartrate" OR Perindopril* OR Phenoxybenzamine OR Phentolamine OR Pinacidil OR Pindolol OR Piperoxan OR Polythiazide OR Prazosin OR Propranolol OR Protoveratrines OR Quinapril* OR Ramipril* OR remikiren OR rentiapril OR Reserpine OR Rilmenidine OR ryodipine OR "saprisartan potassium" OR scoparone OR selexipag OR sesamin OR spirapril OR talinolol OR Telmisartan OR "temocapril hydrochloride" OR Teprotide OR Terlipressin OR tetrahydropalmatine OR theodrenaline OR tibolone OR Ticrynafen OR Timolol OR tobanum OR tocopherylquinone OR Todralazine OR Tolazoline OR Torsemide OR trandolapril OR Travoprost OR treprostinil OR Trichlormethiazide OR trimazosin OR Trimethaphan OR urapidil OR Valsartan OR "Veratrum Alkaloids" OR Vincamine OR viprostol OR Xipamide OR "Y 26763" OR "Y 27632" OR zofenopril)	
Blood pressure reduction (combined)	6	S4 OR S5	64,698
Cognition (Controlled vocabulary)	7	MH (Cognition OR "Cognition Disorders" OR "Mild Cognitive Impairment" OR "Dementia+" OR Memory OR "Alzheimer's Disease")	160,623
Cognition (Free vocabulary)	8	TI (Cogn* OR dement* OR neurocogn* OR alzheimer* OR (mental N2 (Disorders OR decline OR impairment OR Deterioration OR syndromes OR Dysfunction))) OR AB (Cogn* OR dement* OR neurocogn* OR alzheimer* OR (mental N2 (Disorders OR decline OR impairment OR Deterioration OR syndromes OR Dysfunction)))	219,805
Cognition (combined)	9	S7 OR S8	275,020
Randomized controlled clinical trials search filter	10	MH "Clinical Trials+" OR MW "DT" OR TI randomized OR AB randomized OR TI randomised OR AB randomised OR TI placebo OR AB placebo OR TI randomly OR AB randomly OR TI trial OR AB trial OR TI groups OR AB groups	1,485,116
Combination of concepts	11	S3 AND S6 AND S9 AND S10	720

© 2021 Dallaire-Théroux C et al. *JAMA Network Open.*

f. Web of Science

Date of the search: 27-10-2020
Database limit: No database limit has been applied

Concepts	#	Search strategy	Results
Adult population	1	TS=(Adult$ OR (old* NEAR/2 (people OR person$ OR adult$ OR patient)) OR elder* OR aged OR senior$ OR geriatri* OR "middle age")	4,810,683
Blood pressure reduction	2	TS=(antihypertens* OR "anti-hypertens*" OR diuretic$ OR thiazide$ OR (calcium NEAR/2 (antagonist$ OR Blocker$)) OR "angiotensin converting enzyme inhibitors" OR "ACE Inhibitor$" OR "angiotensin receptor blocker$" OR Acebutolol OR Adrenomedullin OR AE0047 OR alacepril OR Alprenolol OR ambrisentan OR Amlodipine OR amosulalol OR "angiotensin I" OR aprikalim OR Atenolol OR "atrial natriuretic factor prohormone" OR azepexole OR benazepril* OR bendazole OR Bendroflumethiazide OR benoxathian OR Bepridil OR berbamine OR Betaxolol OR Bethanidine OR bietaserpine OR bimakalim OR Bimatoprost OR Bisoprolol OR Bosentan OR "BQ 22-708" OR "BQ 788" OR "Bretylium Tosylate" OR "Brimonidine Tartrate" OR budralazine OR Bupranolol OR buthiazide OR cadralazine OR cafedrine OR candesartan OR candoxatril OR Captopril OR Carteolol OR Carvedilol OR Celiprolol OR ceronapril OR Chlorisondamine OR Chlorothiazide OR Chlorthalidone OR cicletanine OR Cilazapril OR cilazaprilat OR clentiazem OR Clonidine OR Cromakalim OR cyclo OR Cyclopenthiazide OR cyclothiazide OR dauricine OR Debrisoquin OR delapril OR "diallyl disulfide" OR Diazoxide OR Dihydralazine OR Dihydroalprenolol OR Diltiazem OR dorzolamide OR Doxazosin OR efonidipine OR Enalapril* OR epanolol OR Eplerenone OR Epoprostenol OR eprosartan OR "essential 303 forte" OR etozolin OR Felodipine OR Fenoldopam OR "ferulic acid" OR "FK 409" OR flesinoxan OR Fosinopril* OR grayanotoxin I OR Guanabenz OR guanadrel OR Guanethidine OR Guanfacine OR Hexamethonium OR Hydralazine OR Hydrochlorothiazide OR Hydroflumethiazide OR imidapril OR Indapamide OR indenolol OR Indoramin OR indorenate OR Irbesartan OR "isopropyl unoprostone" OR Isradipine OR Kallidin OR Ketanserin OR "L 158809" OR Labetalol OR lacidipine OR Latanoprost OR lercanidipine OR libenzapril OR linsidomine OR Lisinopril OR lofexidine OR Losartan OR manidipine OR Mecamylamine OR medroxalol OR "medullipin I" OR Methyldopa OR Metipranolol OR Metolazone OR Metoprolol OR Mibefradil OR Minoxidil OR moexipril OR monatepil OR moxonidine OR Muzolimine OR Nadolol OR naftopidil OR Nebivolol OR Nicardipine OR Nicorandil OR niguldipine OR nilvadipine OR Nimodipine OR "NIP 121" OR nipradilol OR Nisoldipine OR Nitrendipine OR Nitroprusside OR oleuropein OR olmesartan OR omapatrilat OR Oxprenolol OR Pargyline OR Pempidine OR Penbutolol OR	389,476

© 2021 Dallaire-Théroux C et al. *JAMA Network Open.*

		"Pentolinium Tartrate" OR Perindopril* OR Phenoxybenzamine OR Phentolamine OR Pinacidil OR Pindolol OR Piperoxan OR Polythiazide OR Prazosin OR Propranolol OR Protoveratrines OR Quinapril* OR Ramipril* OR remikiren OR rentiapril OR Reserpine OR Rilmenidine OR ryodipine OR "saprisartan potassium" OR scoparone OR selexipag OR sesamin OR spirapril OR talinolol OR Telmisartan OR "temocapril hydrochloride" OR Teprotide OR Terlipressin OR tetrahydropalmatine OR theodrenaline OR tibolone OR Ticrynafen OR Timolol OR tobanum OR tocopherylquinone OR Todralazine OR Tolazoline OR Torsemide OR trandolapril OR Travoprost OR treprostinil OR Trichlormethiazide OR trimazosin OR Trimethaphan OR urapidil OR Valsartan OR "Veratrum Alkaloids" OR Vincamine OR viprostol OR Xipamide OR "Y 26763" OR "Y 27632" OR zofenopril)	
Cognition	3	TS=(Cogn* or dement* OR neurocogn* OR alzheimer* OR (mental NEAR/2 (Disorders OR decline OR impairment OR Deterioration OR syndromes OR Dysfunction)))	1,085,140
Randomized controlled clinical trials search filter	4	TS=(randomized OR randomised OR placebo OR randomly OR trial OR groups OR "drug therapy")	6,700,023
Combination of concepts	5	#1 AND #2 AND #3 AND #4	1,228

© 2021 Dallaire-Théroux C et al. *JAMA Network Open.*

eTable 2. Additional Demographic Characteristics of Studies Included in Quantitative Analyses

Trial (year)	Mean age (years, SD)	Female (%)	Caucasians (%)	Smokers (%)	Hypertension (%)	Diabetes mellitus (%)	CKD (%)	CAD (%)	Cognitively healthy (%)	MCI (%)	Prevention of cognitive decline	Prevention of stroke
ACCORD BP (2010) [27, 28]	62.2 (6.9)	47.7	60.5	55.1	N/A	100.0	N/A	33.7	N/A (ACCORD-MIND : 100.0)	N/A (ACCORD-MIND : 0.0)	Mixed (ACCORD-MIND: primary prevention)	Mixed
SPS3 (2013) [29, 30]	63.0 (11)	37.0	51.0	20.0	75.0	37.0	N/A	11.0	55.3	44.7	Mixed	Secondary prevention
SPRINT (2015) [15, 31-34]	67.9 (9.5)	35.6	57.7	55.7	100.0	0.0	28.3	20.1	N/A	N/A	Mixed	Primary prevention
PODCAST (2017) [35-37]	74.0 (6.8)	22.9	N/A	67.5	83.1	20.5	N/A	24.1	55.8	44.2	Mixed	Secondary prevention
INFINITY (2019) [38, 39]	80.5 (4.1)	54.0	87.0	60.8	100.0	16.0	23.0	39.0	N/A	N/A	Mixed	Primary prevention

Abbreviations: CAD, coronary artery disease; CKD, chronic kidney disease; MCI, mild cognitive impairment; N/A, not available; SD, standard deviation.

© 2021 Dallaire-Théroux C et al. *JAMA Network Open.*

eTable 3. Subgroup Analysis for the Outcome of Cognitive Decline

Outcome	Subgroups	Trials	Participants (intensive/standard)	Weight	Effect Estimate (95% CI)	I^2
Cognitive decline	Total	4	2606/2640	100.0%	SMD 0.01 (-0.04, 0.06)	0%
	Follow-up					
	Up to 3 years	2	439/447	16.9%	SMD 0.09 (-0.04, 0.22)	0%
	More than 3 years	2	2167/2193	83.1%	SMD -0.01 (-0.07, 0.05)	0%
	Age					
	< 65 years old	2	1091/1151	42.7%	SMD 0.02 (-0.08, 0.12)	25%
	> 65 years old	2	1515/1489	57.3%	SMD 0.01 (-0.07, 0.08)	0%
	Diabetes					
	Diabetic patients	1	745/694	27.4%	SMD 0.02 (-0.08, 0.12)	-
	Non-diabetic patients	1	1473/1448	55.7%	SMD 0.00 (-0.07, 0.07)	-
	Mixed	2	439/447	16.9%	SMD 0.09 (-0.04, 0.22)	0%
	Prevention of cognitive decline					
	Primary prevention	1	694/745	27.4%	SMD -0.02 (-0.12, 0.08)	-
	Secondary prevention	0	0/0	0%	-	-
	Mixed	3	1912/1895	72.6%	SMD 0.02 (-0.04, 0.08)	0%
	Prevention of stroke					
	Primary prevention	1	1473/1448	55.7%	SMD 0.00 (-0.07, 0.07)	-
	Secondary prevention	2	439/447	16.9%	SMD 0.09 (-0.04, 0.22)	0%
	Mixed	1	694/745	27.4%	SMD -0.02 (-0.12, 0.08)	-
	Risk of bias					
	Low risk of bias	0	0/0	0%	-	-
	Unclear risk of bias	4	2606/2640	100.0%	SMD 0.01 (-0.04, 0.06)	0%
	High risk of bias	0	0/0	0%	-	-

Abbreviations: CI, confidence interval; SMD, standardized mean difference.

© 2021 Dallaire-Théroux C et al. *JAMA Network Open.*

eTable 4. Subgroup Analysis for the Outcome of Probable Dementia

Outcome	Subgroups	Trials	Participants (intensive/standard)	Weight	Effect Estimate (95% CI)	I^2
Probable dementia	Total	2	4719/4725	100.0%	RR 1.09 (0.32, 3.67)	27%
	Follow-up					
	Up to 3 years	1	41/42	13.8%	RR 5.12 (0.25, 103.48)	-
	More than 3 years	1	4678/4683	86.2%	RR 0.85 (0.68, 1.05)	-
	Age					
	< 65 years old	0	0/0	0%	-	-
	> 65 years old	2	4719/4725	100.0%	RR 1.09 (0.32, 3.67)	27%
	Diabetes					
	Diabetic patients	0	0/0	0%	-	-
	Non-diabetic patients	1	4678/4683	86.2%	RR 0.85 (0.68, 1.05)	-
	Mixed	1	41/42	13.8%	RR 5.12 (0.25, 103.48)	-
	Prevention of cognitive decline					
	Primary prevention	0	0/0	0%	-	-
	Secondary prevention	0	0/0	0%	-	-
	Mixed	2	4719/4725	100.0%	RR 1.09 (0.32, 3.67)	27%
	Prevention of stroke					
	Primary prevention	1	4678/4683	86.2%	RR 0.85 (0.68, 1.05)	-
	Secondary prevention	1	41/42	13.8%	RR 5.12 (0.25, 103.48)	-
	Mixed	0	0/0	0%	-	-
	Risk of bias					
	Low risk of bias	0	0/0	0%	-	-
	Unclear risk of bias	2	4719/4725	100.0%	RR 1.09 (0.32, 3.67)	27%
	High risk of bias	0	0/0	0%	-	-

Abbreviations: CI, confidence interval; RR, risk ratio.

© 2021 Dallaire-Théroux C et al. *JAMA Network Open.*

eTable 5. Subgroup Analysis for the Outcome of Mild Cognitive Impairment

Outcome	Subgroups	Trials	Participants (intensive/standard)	Weight	Effect Estimate (95% CI)	I^2
Mild cognitive impairment	Total	2	5391/5383	100.0%	RR 0.91 (0.73, 1.14)	74%
	Follow-up					
	Up to 3 years	1	713/700	48.2%	RR 1.02 (0.86, 1.22)	-
	More than 3 years	1	4678/4683	51.8%	RR 0.81 (0.70, 0.95)	-
	Age					
	< 65 years old	1	713/700	48.2%	RR 1.02 (0.86, 1.22)	-
	> 65 years old	1	4678/4683	51.8%	RR 0.81 (0.70, 0.95)	-
	Diabetes					
	Diabetic patients	0	0/0	0%	-	-
	Non-diabetic patients	1	4678/4683	51.8%	RR 0.81 (0.70, 0.95)	-
	Mixed	1	713/700	48.2%	RR 1.02 (0.86, 1.22)	-
	Prevention of cognitive decline					
	Primary prevention	0	0/0	0%	-	-
	Secondary prevention	0	0/0	0%	-	-
	Mixed	2	5391/5383	100.0%	RR 0.91 (0.73, 1.14)	74%
	Prevention of stroke					
	Primary prevention	1	4678/4683	51.8%	RR 0.81 (0.70, 0.95)	-
	Secondary prevention	1	713/700	48.2%	RR 1.02 (0.86, 1.22)	-
	Mixed	0	0/0	0%	-	-
	Risk of bias					
	Low risk of bias	0	0/0	0%	-	-
	Unclear risk of bias	0	0/0	0%	-	-
	High risk of bias	2	5391/5383	100.0%	RR 0.91 (0.73, 1.14)	74%

Abbreviations: CI, confidence interval; RR, risk ratio.

© 2021 Dallaire-Théroux C et al. *JAMA Network Open.*

eTable 6. Subgroup Analysis for the Outcome of Cerebrovascular Events

Outcome	Subgroups	Trials	Participants (intensive/standard)	Weight	Effect Estimate (95% CI)	I^2
Cerebrovascular events	Total	5	8681/8715	100.0%	RR 0.79 (0.67, 0.93)	0%
	Follow-up					
	Up to 3 years	2	140/142	1.1%	RR 0.41 (0.08, 2.08)	0%
	More than 3 years	3	8541/8573	98.9%	RR 0.78 (0.63, 0.97)	28%
	Age					
	< 65 years old	2	3863/3890	73.9%	RR 0.73 (0.52, 1.02)	56%
	> 65 years old	3	4818/4825	26.1%	RR 0.86 (0.62, 1.20)	0%
	Diabetes					
	Diabetic patients	1	2362/2371	17.4%	RR 0.58 (0.39, 0.88)	-
	Non-diabetic patients	1	4678/4683	25.0%	RR 0.89 (0.63, 1.24)	-
	Mixed	3	1641/1661	57.6%	RR 0.82 (0.66, 1.03)	0%
	Prevention of cognitive decline					
	Primary prevention	0	0/0	0%	-	-
	Secondary prevention	0	0/0	0%	-	-
	Mixed	5	8681/8715	100.0%	RR 0.79 (0.67, 0.93)	0%
	Prevention of stroke					
	Primary prevention	2	4777/4783	25.5%	RR 0.88 (0.63, 1.23)	0%
	Secondary prevention	2	1542/1561	57.1%	RR 0.82 (0.66, 1.03)	0%
	Mixed	1	2362/2371	17.4%	RR 0.58 (0.39, 0.88)	-
	Risk of bias					
	Low risk of bias	0	0/0	0%	-	-
	Unclear risk of bias	4	8582/8615	99.5%	RR 0.78 (0.65, 0.95)	10%
	High risk of bias	1	99/100	0.5%	RR 0.51 (0.05, 5.48)	-

Abbreviations: CI, confidence interval; RR, risk ratio.

© 2021 Dallaire-Théroux C et al. *JAMA Network Open.*

eTable 7. Subgroup Analysis for the Outcome of Serious Adverse Events

Outcome	Subgroups	Trials	Participants (intensive/standard)	Weight	Effect Estimate (95% CI)	I^2
Serious adverse events	Total	5	8681/8715	100.0%	RR 1.13 (0.91, 1.40)	65%
	Follow-up					
	Up to 3 years	2	140/142	32.4%	RR 0.91 (0.69, 1.20)	0%
	More than 3 years	3	8541/8573	67.6%	RR 1.29 (0.91, 1.81)	80%
	Age					
	< 65 years old	2	3863/3890	32.4%	RR 1.55 (1.22, 1.98)	0%
	> 65 years old	3	4818/4825	67.6%	RR 1.03 (0.98, 1.08)	0%
	Diabetes					
	Diabetic patients	1	2362/2371	23.7%	RR 1.55 (1.19, 2.02)	-
	Non-diabetic patients	1	4678/4683	35.2%	RR 1.03 (0.98, 1.09)	-
	Mixed	3	1641/1661	41.1%	RR 1.00 (0.75, 1.34)	17%
	Prevention of cognitive decline					
	Primary prevention	0	0/0	0%	-	-
	Secondary prevention	0	0/0	0%	-	-
	Mixed	5	8681/8715	100.0%	RR 1.13 (0.91, 1.40)	65%
	Prevention of stroke					
	Primary prevention	2	4777/4783	53.4%	RR 1.03 (0.98, 1.09)	0%
	Secondary prevention	2	1542/1561	22.9%	RR 1.09 (0.60, 1.98)	57%
	Mixed	1	2362/2371	23.7%	RR 1.55 (1.19, 2.02)	-
	Risk of bias					
	Low risk of bias	0	0/0	0%	-	-
	Unclear risk of bias	4	8582/8615	81.9%	RR 1.18 (0.89, 1.55)	73%
	High risk of bias	1	99/100	18.1%	RR 0.96 (0.67, 1.37)	-

Abbreviations: CI, confidence interval; RR, risk ratio.

© 2021 Dallaire-Théroux C et al. *JAMA Network Open.*

eTable 8. Subgroup Analysis for the Outcome of All-Cause Mortality

Outcome	Subgroups	Trials	Participants (intensive/standard)	Weight	Effect Estimate (95% CI)	I^2
All-cause mortality	Total	5	8681/8715	100.0%	RR 0.93 (0.75, 1.15)	48%
	Follow-up					
	Up to 3 years	2	140/142	3.8%	RR 0.90 (0.30, 2.66)	0%
	More than 3 years	3	8541/8573	96.2%	RR 0.93 (0.73, 1.19)	71%
	Age					
	< 65 years old	2	3863/3890	61.5%	RR 1.05 (0.89, 1.25)	0%
	> 65 years old	3	4818/4825	38.5%	RR 0.74 (0.61, 0.91)	0%
	Diabetes					
	Diabetic patients	1	2362/2371	32.8%	RR 1.05 (0.84, 1.30)	-
	Non-diabetic patients	1	4678/4683	34.7%	RR 0.74 (0.60, 0.91)	-
	Mixed	3	1641/1661	32.5%	RR 1.05 (0.81, 1.36)	0%
	Prevention of cognitive decline					
	Primary prevention	0	0/0	0%	-	-
	Secondary prevention	0	0/0	0%	-	-
	Mixed	5	8681/8715	100.0%	RR 0.93 (0.75, 1.15)	48%
	Prevention of stroke					
	Primary prevention	2	4777/4783	36.3%	RR 0.73 (0.60, 0.90)	0%
	Secondary prevention	2	1542/1561	30.9%	RR 1.07 (0.83, 1.39)	0%
	Mixed	1	2362/2371	32.8%	RR 1.05 (0.84, 1.30)	-
	Risk of bias					
	Low risk of bias	0	0/0	0%	-	-
	Unclear risk of bias	4	8582/8615	98.4%	RR 0.94 (0.75, 1.18)	58%
	High risk of bias	1	99/100	1.6%	RR 0.51 (0.09, 2.69)	-

Abbreviations: CI, confidence interval; RR, risk ratio.

© 2021 Dallaire-Théroux C et al. *JAMA Network Open.*

eFigure. Sensitivity Analysis Using Fixed-Effect Model for the Incidence of Probable Dementia

Study or Subgroup	Intensive Events	Intensive Total	Standard Events	Standard Total	Weight	Risk Ratio IV, Fixed, 95% CI	Year
SPRINT 16,31-34 2015	149	4678	176	4683	99.5%	0.85 [0.68, 1.05]	2015
PODCAST 35-37 2017	2	41	0	42	0.5%	5.12 [0.25, 103.48]	2017
**Total (95% CI)**		**4719**		**4725**	**100.0%**	**0.86 [0.69, 1.06]**	
Total events	151		176				
Heterogeneity: Chi² = 1.37, df = 1 (P = 0.24); I² = 27%							
Test for overall effect: Z = 1.43 (P = 0.15)							

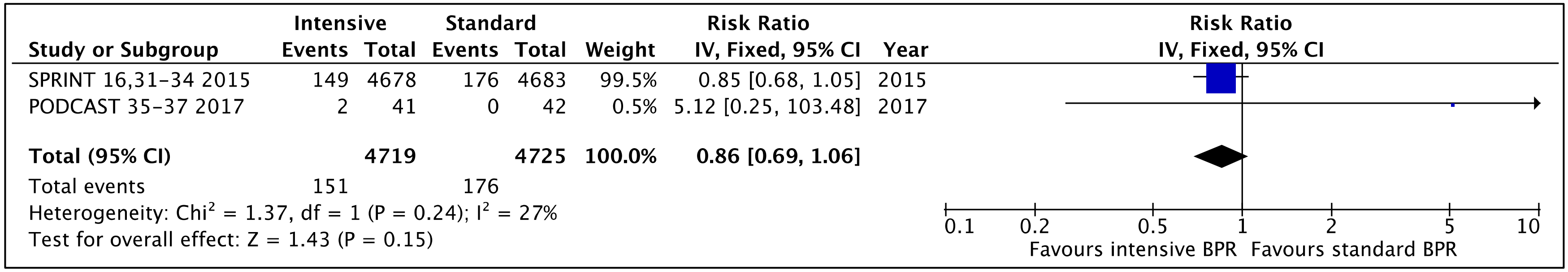

Abbreviations: BPR, blood pressure reduction; CI, confidence interval; IV, inverse variance.

The squares and bars represent the mean values and 95% CIs of the effect sizes. The area of the squares indicates the weight of each individual study. The diamond represents the combined effect in pooled studies and the vertical line represents the null value (no association).

© 2021 Dallaire-Théroux C et al. *JAMA Network Open.*
